# In Situ Eutectic Formation in a Polymeric Matrix via Hot-Melt Reactive Extrusion and the Use of Partial Least Squares Regression Modeling for Reaction Yield Determination

**DOI:** 10.1021/acs.molpharmaceut.4c00152

**Published:** 2024-08-13

**Authors:** Gavin P. Andrews, Alice Culkin, David S. Jones, Shu Li

**Affiliations:** The Pharmaceutical Engineering Group, School of Pharmacy, Queen’s University Belfast, 97 Lisburn Road, Belfast, Northern Ireland BT9 7BL, U.K.

**Keywords:** reactive extrusion, mechanochemistry, albendazole, eutectic, multivariate analysis, partial least-squares regression

## Abstract

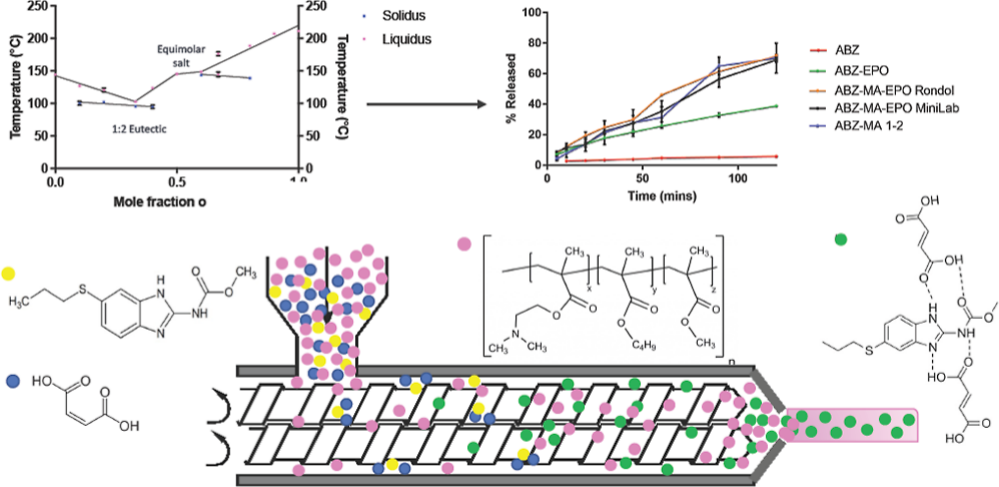

There has been a significant volume of work investigating the design and synthesis of new crystalline multicomponent systems via examining complementary functional groups that can reliably interact through the formation of noncovalent bonds, such as hydrogen bonds (H-bonds). Crystalline multicomponent molecular adducts formed using this approach, such as cocrystals, salts, and eutectics, have emerged as drug product intermediates that can lead to effective drug property modifications. Recent advancement in the production for these multicomponent molecular adducts has moved from batch techniques that rely upon intensive solvent use to those that are solvent-free, continuous, and industry-ready, such as reactive extrusion. In this study, a novel eutectic system was found when processing albendazole and maleic acid at a 1:2 molar ratio and successfully prepared using mechanochemical methods including liquid-assisted grinding and hot-melt reactive extrusion. The produced eutectic was characterized to exhibit a 100 °C reduction in melting temperature and enhanced dissolution performance (>12-fold increase at 2 h point), when compared to the native drug compound. To remove handling of the eutectic as a formulation intermediate, an end-to-end continuous-manufacturing-ready process enables feeding of the raw parent reagents in their respective natural forms along with a chosen polymeric excipient, Eudragit EPO. The formation of the eutectic was confirmed to have taken place in situ in the presence of the polymer, with the reaction yield determined using a multivariate calibration model constructed by combining spectroscopic analysis with partial least-squares regression modeling. The ternary extrudates exhibited a dissolution profile similar to that of the 1:2 prepared eutectic, suggesting a physical distribution (or suspension) of the in situ synthesized eutectic contents within the polymeric matrix.

## Introduction

One of the most effective methods of altering the physicochemical properties of an active pharmaceutical ingredient (API) is the formation of a multicomponent crystal (MCC). An MCC is formed when there are noncovalent, and hence supramolecular, interactions occurring between the components which make up the bonded system, where a component may be defined as either an atom, ion, or molecule.^1^ Crystal engineering is a common method used in order to efficiently design and produce these MCCs based on the supramolecular synthon approach. MCCs formed using this approach have also emerged as an effective strategy to improve the bioavailability of poorly soluble drug compounds without having to compromise on thermodynamic stability.

**Figure 1:**
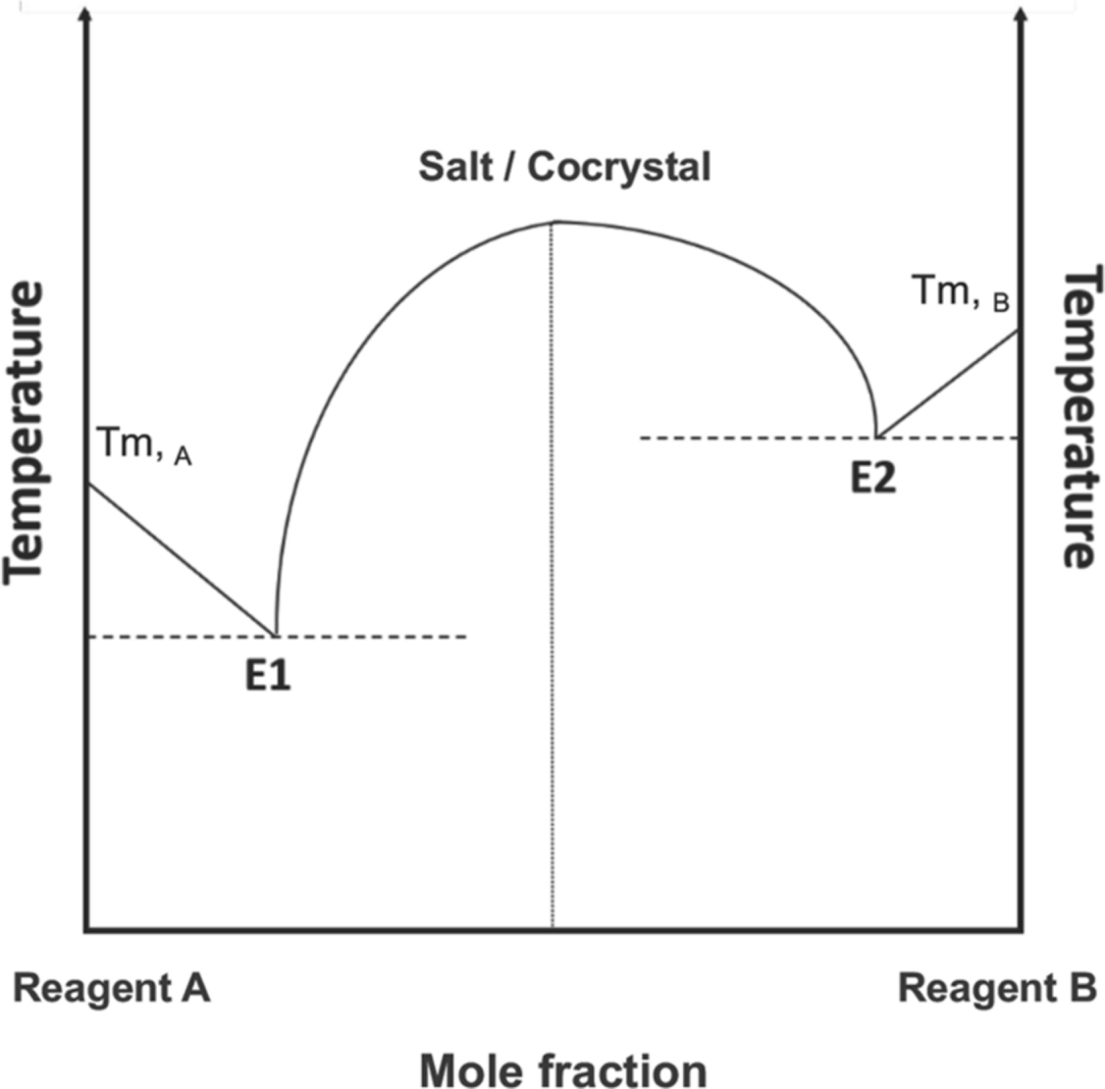
Typical phase diagram of a binary salt or cocrystal, showing the point of salt/cocrystal formation and two eutectic points (E1 and E2).

Pharmaceutical salts are the most common pharmaceutical MCC, with it being estimated that more than 50% of APIs are being administered in salt form.^2^ Salts are crystalline materials, where proton transfer has taken place between an acid and a base; these generally form when the Δp*K*_a_ between acid and the protonated base is >3.0.^3^ Where the counterion in a pharmaceutical salt is an organic compound, the respective ions in the salt are typically bound together through protonation and the formation of ionic bonds. Dependent upon the extent of protonation, a fine line lies between whether an MCC is a salt or a cocrystal. The thermodynamic phase diagram of a typical binary salt or cocrystal usually exhibits a classic “W” shape, where the salt or cocrystal is seen to form at a defined stoichiometric ratio, with two low-melting eutectic points (E1 and E2) on either side of the salt/cocrystal forming point (Figure 1). These eutectic compositions generally consist of mixtures of either both parent components or mixtures of the formed eutectic with one of the parent components that is in excess and melt at a lower temperature than the binary system.^4^ Since the eutectic composition between the lower-melting parent and the salt/cocrystal is likely to exhibit the lowest melting temperature among all components of the system, formation of this eutectic may also present itself as a potential strategy for the processing of high-melting or thermolabile APIs.

Traditionally, pharmaceutical salts and cocrystals have always been treated as pharmaceutical product intermediates and manufactured using methods which are extremely solvent-intensive,^5^ followed by multiple-stepped purification procedures, in order to achieve high levels of purity. However, due to reliance on organic solvents where drugs with poor aqueous solubility are concerned, these processes are being increasingly considered as wasteful and harmful to the environment.^6^ As a result, there is a drive to use processes that require little or no solvent. Mechanochemical manufacturing, using techniques such as liquid-assisted grinding (LAG) and hot-melt reactive extrusion (HMRE), has been proven as a viable method for the synthesis of pharmaceutical salts and cocrystals.^7−9^ Similar to the addition of a small amount of solvent to the grinding process, which is known to facilitate bonding between the API and the guest molecule,^10^ the presence of a suitable nonsolvent matrix excipient has also been proven to improve both the kinetics and yield of the reaction during these mechanochemical processes.^8,9,11^

**Figure 2:**
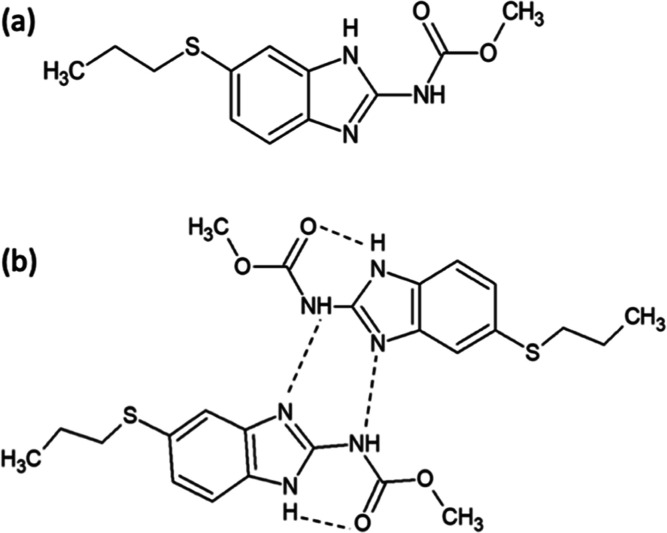
(a) Molecular structure of ABZ and (b) intra- and intermolecular H-bonded network of ABZ. Reproduced from ref (23). Copyright [2015] American Chemical Society.

Albendazole (ABZ, Figure 2a), a BCS Class II broad-spectrum anthelmintic,^12^ is selected as a model compound in this study due to its poor water solubility,^12,13^ high p*K*_a_ (10.26^13^), high melting temperature (208 °C^12^), and the fact that it degrades upon melting. ABZ is derived from a benzimidazole central structure, and it has, in recent years, been gaining increasing attention for its anticancer potential against a variety of tumors, including ovarian^14^ and colorectal^15^ cancer models.

ABZ shows common characteristics of a “brick dust” molecule, with extremely poor aqueous solubility (0.2 pg/mL at 25 °C) as a result of its extensive intramolecular and intermolecular H-bonded network (Figure 2b). In particular, the intramolecular H-bond between the imidazole N–H and the carbonyl group is thought to be the primary cause of their particularly low aqueous solubility.^12^ Crystal engineering approaches have been relatively less explored for ABZ, with a much larger volume of work focusing on the preparation of solid dispersions^16−19^ and inclusion complexes using cyclodextrins.^20^ Salts of ABZ have been reported using carboxylic acids such as oxalic acid, tartaric acid, and maleic acid (MA), where it was found that ABZ commonly forms fragile crystals which can be difficult to crystallize.^21^ Other ABZ salts using hydrochloric acid, methanesulfonic acid, sulfuric acid, and *para*-toluene sulfonic acid have also been reported in the literature with improved dissolution behaviors compared to the pure drug.^22^ However, none of these reported salt forms of ABZ are commercially available.

In this paper, MA, a highly water-soluble (440.7 g/L at 25 °C^24^) GRAS dicarboxylic acid, is used as the Brønsted–Lowry acid (or “counterion”) in our attempted eutectic synthesis based on preliminary material screening for successful eutectic formation with ABZ using LAG. However, principles applied on matrix excipient selection focused on their aqueous solubility, processability within HME, as well as the compatibility with ABZ, MA, and any form of the ABZ–MA-bonded system. With these criteria, Eudragit EPO (EPO), a cationic methacrylate polymer with reasonably low *T*_g_ and a rapid dissolution profile in gastric fluids, was promoted from preliminary screening and selected as the polymeric carrier excipient in this work.

It was aimed to obtain the eutectic in isolation from other components of the system so that a profile of its physicochemical properties can be established. To achieve this, LAG was utilized as a small-scale batch-production method to obtain the ABZ–MA salt and eutectic systems, respectively, using mechanochemistry. Following success of the preliminary studies, latter work aimed to explore whether the said eutectic system can be synthesized, in situ, in the presence of EPO using HMRE, so that a single-step, concurrent eutectic synthesis and formulation process may be established for ABZ.

When performing an in situ reaction in a continuous preparation such as HMRE, with limited time frame for the reaction to take place, it is important to understand the reaction kinetics, so that both formulation and processing can be optimized to ensure a high reaction efficiency. To achieve this, the first and foremost step would involve establishing a reliable quantitative assay to determine the reaction yield during and following extrusion. It was, therefore, a third aim of this paper to probe the feasibility of using partial least-squares (PLS) modeling based on multivariate analysis of the spectroscopic data to quantify the content of the formed ABZ–MA eutectic within a matrix of EPO and the eutectic parent reagents.

## Materials and Methods

### Materials

ABZ (purity >98%) was purchased from Kemprotec (Cumbria, United Kingdom). MA (purity >99%) and monobasic ammonium phosphate (purity >98%) were both purchased from Sigma-Aldrich (Gillingham, United Kingdom). Eudragit EPO was a kind gift from Evonik Industries (Essen, Germany). Ultrapure water was obtained using a Direct Q Millipore water purification system. All other chemical reagents used were of analytical grade.

### Liquid-Assisted Grinding

LAG was used here to prepare both the salt and the eutectic mixture between ABZ and MA. ABZ and MA were added to a 50 mL ball milling chamber (CryoMill, Retsch, Germany) either equimolarly (χABZ = 0.50) or in a 1:2 molar ratio (χABZ = 0.33) with two 15 mm stainless steel milling balls. A substoichiometric amount of methanol (η = 0.2 μL/mg) was added to the powder mixtures, prior to closure of the jar with a screw-tight lid. The mixtures were ground for an overall 30 min at 30 Hz, in intervals of 5 min, to reduce the effects of temperature.

### Differential Scanning Calorimetry

Differential scanning calorimetry (DSC) (Thermal Advantage model Q20, TA Instruments, UK) was used to characterize the thermal behavior of all raw ingredients, physical mixtures, and extrudates. The instrument was calibrated by using indium and zinc prior to use. Samples (3–10 mg) were accurately weighed and transferred into aluminum pans which were subsequently crimped with aluminum lids. A heating rate of 10 °C/min was employed, with dry nitrogen used as the purge gas at a flow rate of 50 mL/min. Samples were equilibrated at predetermined temperatures (−65 °C for raw materials and the prepared bonded systems following LAG and 0 °C for formulations following HMRE, respectively) before being heated to 220 °C.

The ABZ–MA thermodynamic binary phase diagram was constructed by plotting the solidus line using the peak temperature of the first melting event and the liquidus line using the peak temperature of the final melting event with respect to each molar ratio tested. Where only one melting event was present, the peak temperature of the single event was treated as the final melting event.

### Powder X-ray Diffraction

Samples were analyzed using a MiniFlex II benchtop X-ray diffractometer (Rigaku) at room temperature using Cu Kα radiation at a voltage of 30 kV and a current of 15 mA. The samples to be tested were placed onto a glass sample holder with a 0.2 mm depression. All samples were scanned within the range 3–45° 2θ in continuous mode with a sampling width of 0.03° and a scan speed of 2.0°/min.

### Attenuated Total Reflectance–Fourier Transform Infrared Spectroscopy (ATR FTIR)

IR spectra were obtained on a PerkinElmer infrared spectrophotometer with a UATR sampling accessory (Spectrum Two FT-IR Spectrometer, PerkinElmer Instruments, USA) over 16 scans with a resolution of 4 cm^–1^ from 4000 to 650 cm^–1^. Data was plotted as transmittance (%) over wavenumber (cm^–1^) and processed using Spectrum IR (PerkinElmer Instruments, USA).

### Hot-Melt Reactive Extrusion

HMRE was carried out by feeding the binary physical mixture of ABZ–EPO or the ternary physical mixture of ABZ–MA–EPO at an equivalent to 10% w/w ABZ into a HAAKE Minilab conical twin screw extruder (Thermo Electron Corporation, Stone, U.K.) equipped with a 2 mm rod die. The processing temperature was maintained at 120 °C, and the screw speed was set to 30 rpm.

A second extruder was also employed to determine how increased intensive mixing may influence the in situ yield of the ABZ–MA eutectic within the EPO matrix. Powder blends of the formulation were fed into a corotating 10 mm Rondol Microlab 20:1 twin screw extruder (Rondol Technology Ltd., Staffordshire, U.K.) equipped with a 2 mm rod die. The screw speed was set at 30 rpm for each formulation, with the material being “starve-fed” into the extruder, to potentially improve mixing by reducing the risk of agglomeration of material.^25^ The temperature was set at 100 °C for the feed zone to allow for sufficient solid conveying immediately after feeding. Zones one to three were set at 120 °C in order to act as the melt zone, and the die temperature was set to 100 °C. The screw profile is detailed in Table 1, incorporating two separate kneading zones.

**Table 1:** Screw Design for HMRE Used on the Rondol Microlab Extruder

screw elements			
feed zone	zone one	zone two	zone three
5 × conveying	3 × 60° mixing2 × conveying	2 × 60° mixing3 × 90° mixing	5 × conveying

### High-Performance Liquid Chromatography

The concentration of ABZ was determined using HPLC-UV. The analysis was carried out using an Agilent HPLC 1200 series, consisting of a binary pump, degasser, column oven, and variable wavelength UV detector, from Agilent Technologies, UK. Samples were analyzed using a Zorbax C_18_ column (100 mm × 4.6 mm with 3.5 μm packing) from Agilent Technologies, UK, at 228 nm. The mobile phase consisted of 4.35 mM monobasic ammonium phosphate, pH 4.8 (40%) and methanol (60%). The flow rate was set constant at 1 mL/min, with the column compartment maintained at 30 °C throughout the entire time frame of all analyses. The obtained chromatograms were analyzed using Agilent OpenLab software.

Using the described assay, a linear relationship (*y* = 33,904*x*) was established between the integral intensity of the identified main analyte peak and ABZ concentration, and it was validated for good specificity, linearity (*R*^2^ > 0.99), inter- and intraday variations, and precision.

### In Vitro Dissolution Study

Blank simulated gastric fluid (blank SGF) was prepared using 0.1 M hydrochloric acid, adjusted to pH 1.2. Each vessel in the dissolution bath contained 900 mL of the blank SGF and was maintained at 37 ± 0.5 °C throughout the experiments, with the USP dissolution apparatus II (paddle) being used at a speed of 50 rpm. Powder-filled hydroxypropyl methylcellulose capsules were used for pure ABZ and ABZ–MA 1:2 eutectic, and each contained the equivalent to 200 mg of ABZ. When carrying out dissolution testing on the extrudates, these were cut into equal lengths of 10 mm using a scalpel.

The capsules or extrudates were then added to the vessels (*n* = 3), and 2 mL of solution was removed from each vessel at specified time points of 5, 10, 20, 30, 45, 60, 90, and 120 min and replaced with 2 mL of prewarmed dissolution medium. Samples were subsequently analyzed using high-performance liquid chromatography (HPLC) after filtration.

### Development of a PLS Calibration Model

Several calibration samples were prepared, containing 0, 30, 50, 70, 90, and 100% weight fractions of the eutectic. The total weight of each sample was 250 mg, with the physical mixture and eutectic being ground in a mortar and pestle for 30 s to ensure adequate mixing. For quantification of the model, midrange IR spectra were collected five times per sample, with each sample being prepared in triplicate (15 spectra per sample), using the method detailed previously.

Prior to applying PLS regression, the data underwent preprocessing through scaling to unit variance (UV) and mean centering to ensure that each variable was viewed by the model as having equal importance^26^ and to improve interpretation of the model by subtracting the average value of each variable from the data.^27^ Score plots using the T scores were applied with Hotelling’s *T*-squared distribution, *T*^2^, used to identify outliers within a data set and is conventionally drawn at the 95% probability level.^27^

The PLS calibration model was constructed using SIMCA 15 software (Umetrics Inc., Sweden) and the obtained FTIR spectra. Several parameters were used to determine the performance and validity of the calibration model, including *R*^2^*X* and *R*^2^*Y* as measures of fit through explaining how much variance is explained with the model and *Q*^2^ as a measure of the predictive ability of the model. As an indication of the accuracy of the model, the root-mean-square error of estimation and the root-mean-squared error of cross-validation (RMSEcv) were also determined. The raw data was pretreated using mean centering and variance scaling automatically using SIMCA, with the first derivative also being applied to the whole spectral range (640–4000 cm^–1^) and the second derivative applied to three separate spectral ranges (3035–3265, 1500–1675, and 650–735 cm^–1^).

The method was validated using four samples of known eutectic percentages (20, 40, 60, and 80% eutectic physically mixed with the respective polymer), which were not used in the calibration of the model.

### Statistical Analysis

Results from the dissolution study were statistically evaluated using a Student *t*-test (GraphPad Prism 7.00), where the level of significance was denoted as *P* < 0.05.

## Results and Discussion

Among several types of MCCs, pharmaceutical eutectics have been popularized alongside pharmaceutical cocrystals and have been proposed either as intermediate materials with which to form cocrystals or as useful materials in their own right.^28^ Eutectics have been shown to offer various advantages as drug-containing noncovalently bonded systems that improve physicochemical properties and/or bioperformance, such as aqueous solubility, for challenging APIs. Owing to the high thermodynamic functions, as a result of the crystalline nature, typical eutectic materials exhibit good thermodynamic stabilities^29^ and hence present themselves as a competitive alternative drug enabling strategy, compared to other extensively studied techniques such as drug amorphization.

The prediction of whether a eutectic will form can be difficult, and successful synthesis of these systems is commonly down to a trial-and-error approach. A eutectic may form as the result of a failed cocrystallization experiment, where the complementary interactions that were predicted to occur based on the supramolecular synthon approach do not materialize, due to factors such as mismatched size, geometric shape, or symmetry, between the two starting components. Where the cohesive interactions dominate over adhesive attractions between the molecules of the two parent reagents, the product is likely to be a eutectic.^29^

With this work, we were keen to probe the feasibility of making an ABZ eutectic with enhanced solubility and dissolution performance, to understand key characteristics of the formed eutectic system, and to explore the feasibility of using advanced manufacturing methods to produce this eutectic and its formulations.

### Identification of the ABZ–MA Binary Systems

**Figure 3:**
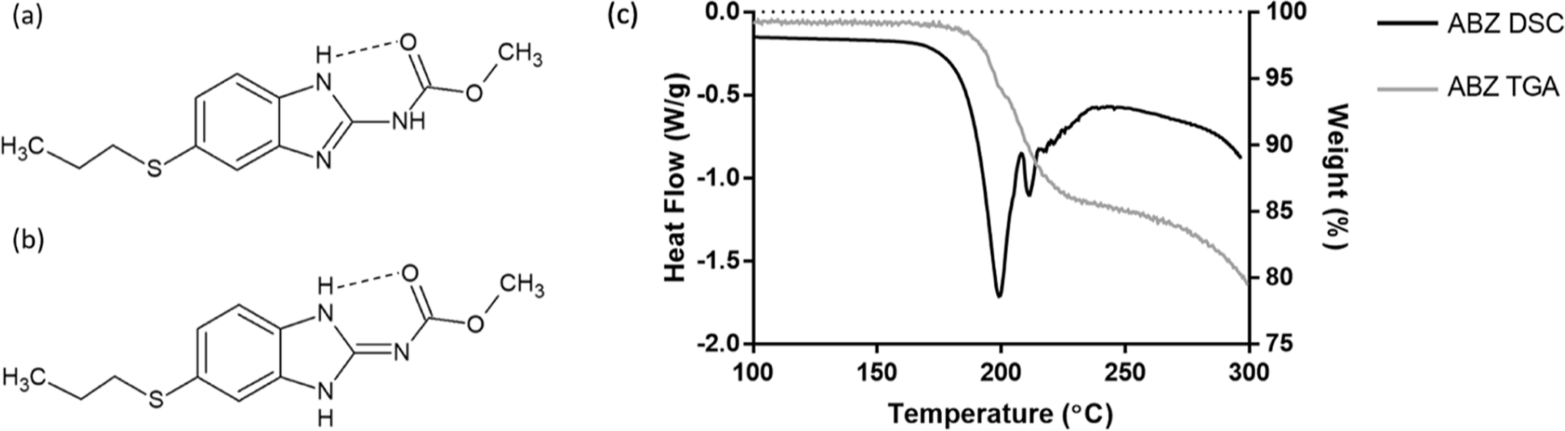
Structure of ABZ tautomers, forms I (a) and II (b), respectively, and the DSC and TGA thermograms of ABZ, with both experiments carried out at 10 °C/min. Chemical structures shown in (a,b) are adapted from ref (30). Copyright [2015] American Chemical Society.

ABZ has been reported to exhibit desmotropy, with two tautomers coexisting and both isolated in the solid state (Figure 3a,b).^30^ The DSC thermogram of ABZ obtained during our material characterizations showed a bimodal melting event with the main melt occurring at 200.98 ± 2.19 °C, followed by a subtle second melt at 211.23 ± 0.37 °C (Figure 3c), agreeing with the reported enantiotropical behaviors of the ABZ isomers.^31^ The TGA thermogram of ABZ exhibited the thermal instability of the drug compound at its *T*_m_. ABZ sensitivity to thermal treatment has been previously reported and attributed to oxidation and production of ABZ sulfoxide at elevated temperatures.^32^

MA, on the other hand, exhibited a single melting peak at 144.3 ± 0.2 °C, which was, however, followed by immediate decomposition prior to reaching the end point of the melting event (Supporting Information Figure 1, χ_ΑΒΖ_ = 0.00). Following mechanochemical treatment using LAG, the ABZ–MA binary systems presented at least one endothermic event. In particular, at ABZ–MA molar ratios of 1:9 (χ_ΑΒΖ_ = 0.10) and 1:2 (χ_ΑΒΖ_ = 0.33), an endothermic peak at approximately 100 °C was observed (100.3 ± 2.9 and 102.3 ± 2.0 °C, respectively). This peak was not, however, present with increasing proportions of ABZ. Latter melting events at these two ratios were detected between 120 and 140 °C, immediately followed by the evidence of thermal instabilities, suggesting depressed *T*_m_ of MA, with no further thermal event from approximately 180 °C onward, implying the absence of ABZ *T*_m_. At an equimolar ratio (χ_ΑΒΖ_ = 0.50), one single melting event was detected at 145.2 ± 0.7 °C, coinciding with a reported *T*_m_ “between 145 and 147 °C” for an ABZ–MA equimolar salt previously reported in the literature.^21^ Further increase of the ABZ content resulted in a broadly depressed ABZ *T*_m_ peak, suggesting that ABZ was in excess (Supporting Information Figure 1).

**Figure 4:**
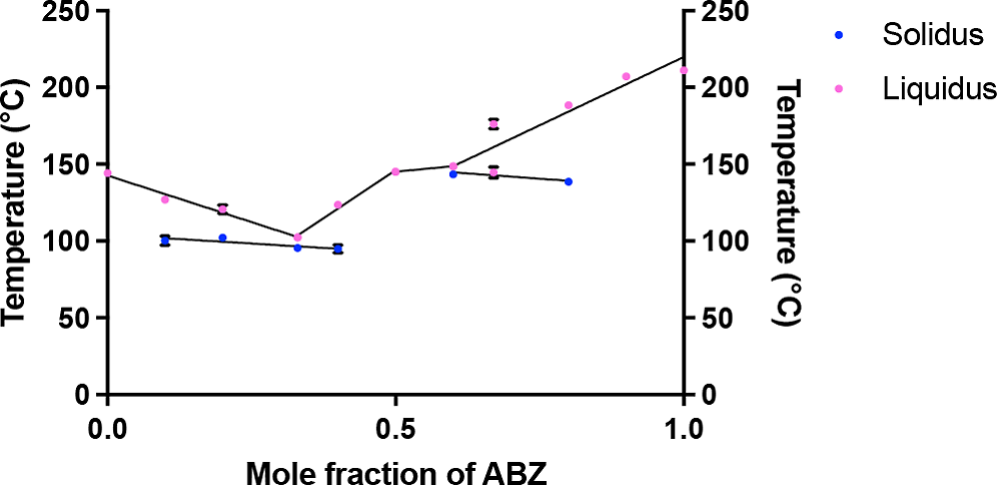
Phase diagram of the ABZ–MA system using the data obtained from the DSC thermograms.

Plotting the melting events observed in the DSC thermograms using the *T*_m_ of the first melting as the solidus (boundary between complete solid and the mixture of solid and melt) and that of the final melting as the liquidus (boundary between the solid/melt mixture and complete melt),^33^ a binary thermodynamic phase diagram may be obtained (Figure 4). It can be observed that the plotted phase diagram exhibited a “W” shape, where, at the equimolar composition, χ_ΑΒΖ_ = 0.50, an ABZ–MA 1:1 salt^21^ was likely the dominant, if not the only, species present in the system. The E1 point was seen to land on χ_ΑΒΖ_ = 0.33 (equivalent to a 1:2 molar ratio between ABZ and MA), whereas the E2 point was likely at χ_ΑΒΖ_ = 0.60.

### Characterizations of the ABZ–MA Salt and Eutectic

#### Solid-State Characterizations

LAG was employed to confirm the feasibility of producing the equimolar ABZ–MA salt and the 1:2 ABZ–MA eutectic in isolation. Preliminary trials have confirmed that a 20 min milling at 22 Hz was sufficient to generate distinguishable salt and the eutectic, with mixed ABZ–MA blends at 1:1 and 1:2, respectively (Figure 6), whereas a prolonged milling duration to 30 min, or a combination of slightly prolonged milling (>25 min) and increased milling frequency (30 Hz), could achieve a 100% 1:2 eutectic yield (Supporting Information Figure 2, percentage yield determined using a validated FTIR/PLS model by employing the full range spectra, 650–4000 cm^–1^, for multivariate analysis).

**Figure 5:**
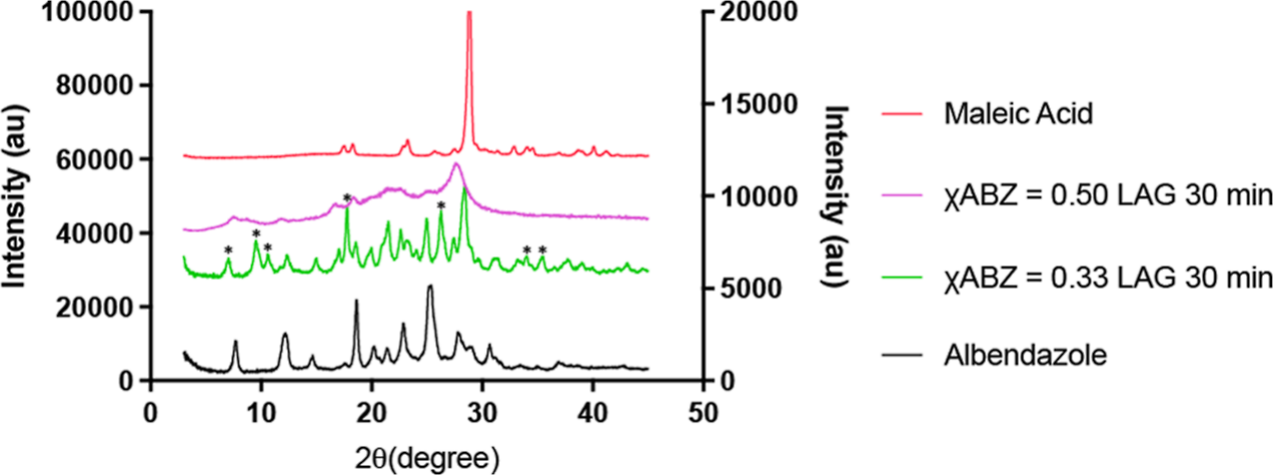
PXRD diffractograms for ABZ, MA, and mixtures of ABZ–MA at χABZ = 0.50 and 0.33, respectively, after LAG for 30 min at 30 Hz. The unique diffraction peaks observed in the ball-milled χABZ = 0.33 are labeled with asterisks. (To enhance visibility of all diffraction peaks, MA was plotted against the left *y*-axis, whereas ABZ and the ball-milled samples were plotted against the right *y*-axis.)

It can be observed from the powder X-ray diffraction (PXRD) patterns (Figure 5) that the pure ABZ exhibited peaks at 7.8, 12.4, 14.8, 18.7, 20.4, 21.6, 23.0, 25.5, 28.0, 29.3, and 30.8° 2θ, characteristic of ABZ form I,^23^ whereas pure MA presented an extremely intense characteristic peak at 28.9° 2θ, with smaller peaks scattered at 18.2, 18.5, 23.4, 33.2, 34.8, 35.0, and 40.0° 2θ, respectively, suggesting a preferred crystallite orientation within the native MA powders. In the PXRD pattern of the χABZ = 0.50 (1–1) blend prepared using LAG, peaks at 7.7, 12.3, 15.2, 18.9, and 21.3° 2θ were able to be identified. It can be seen that the baseline of the χABZ = 0.50 (1–1) blend pattern was considerably raised, potentially suggesting the presence of amorphous contents. This would not be surprising given the fact that the blend was produced using LAG. Because of poor resolution of the obtained PXRD patterns for this product, it was difficult to identify any occurrence of new peaks unique to the parent reagents, ABZ and MA, respectively. As the χABZ = 0.50 (1–1) blend showed the same *T*_m_ as the reported ABZ–MA equimolar salt, the PXRD of the reported salt was used to help identify characteristic peaks of the salt. Although data are only presented in Supporting Information, the reported salt seemed to have presented unique PXRD peaks, to both parent reagents, throughout the 2θ region from 7 to 30°, including split peaks in the 2θ regions of 7–10, 11–12, and 22–24° 2θ, respectively; new peaks appear at approximately 14°, in the regions of 15–20 and 26–27°,^14^ when compared to the ABZ and MA patterns in Figure 5.

With the χABZ = 0.33 (1–2) blend, a comparison with the referenced salt’s PXRD pattern suggested superimposable patterns throughout the majority of the presented 2θ scale. Surprisingly, the eutectic also exhibited two additional new peaks, at 9.7 and 10.7° 2θ, respectively, that did not belong to either parent reagents or the equimolar salt. While whether the two additional peaks indicate new chemical entities will be further examined in a following-up study, it is worth noting that ABZ form II has been reported to exhibit unique PXRD characteristics within several 2θ regions including a peak at 10.5° 2θ.^35^ It is possible that ABZ polymorphic changes had occurred during processing at the χABZ = 0.33 (1–2) composition.

**Figure 6:**
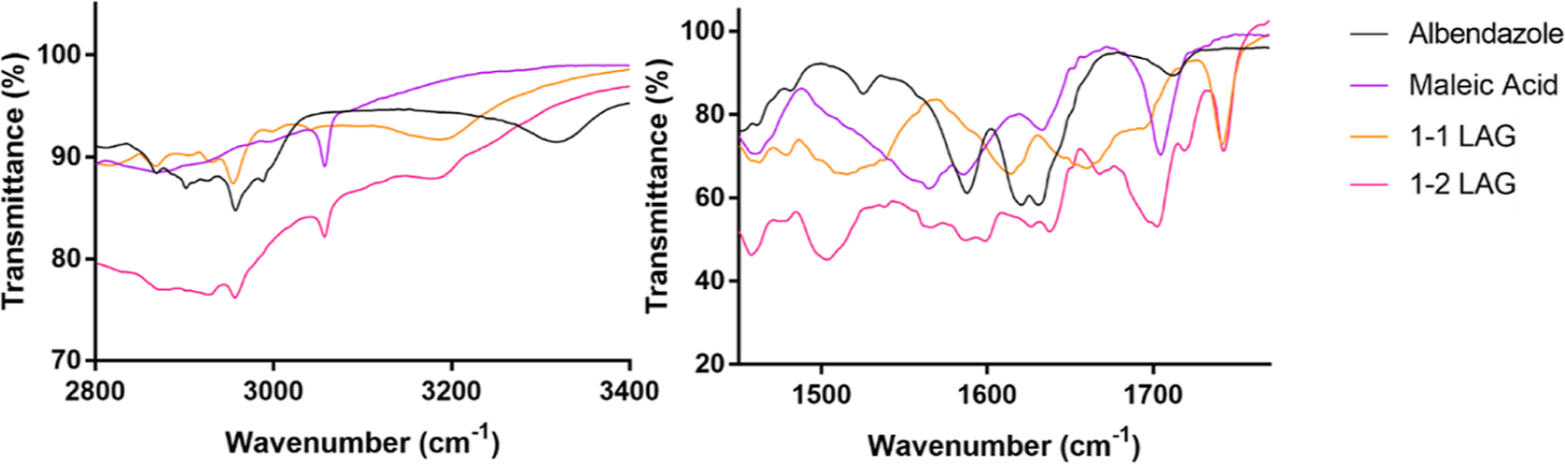
FTIR spectra for ABZ, MA, and ABZ–MA χABZ = 0.50 and 0.33 in the regions of 3400–2800 and 1770–1450 cm^–1^.

Spectroscopic analysis using FTIR was able to reveal the differences within both the amine and carbonyl regions between the 1:2 eutectic and the equimolar salt (Figure 6). ABZ contains amide ester-based functionality and therefore displayed prominent absorption bands at 3320 (broad), 1713 (medium), 1631 (strong), and 1620 (strong) cm^–1^, attributable to stretches of the N–H, the ester C=O, and the amide C=O, respectively. The broad N–H stretch with a peak at 3320 cm^–1^ represented a combined –NH– band from both the amide –NH– and the secondary –NH– from the imidazole ring. Due to the lack of proton surrounding the tertiary amine on the imidazole ring, this amine could not be detected through FTIR. Within the carbonyl region, the medium peak at 1713 cm^–1^ was typical of an ester C=O stretching, whereas the doublet at 1620 and 1631 cm^–1^, respectively, denoted significantly red-shifted ABZ amide C=O stretching, likely due to the intramolecular interactions within each respective ABZ tautomer (Figure 3). Another strong band at 1587 cm^–1^ should be attributed to the tertiary amine –C=N– stretch. MA, on the other hand, presented a strong peak at 1704 cm^–1^ as H-bonded C=O stretching, because MA, like most carboxylic acid compounds, exists in dimeric form through a double carboxylic acid homosynthon in its natural state.^34^

Following LAG, both the 1:2 and equimolar systems exhibited a new band at 1741 cm^–1^, suggesting the presence of carbonyl in a non-H-bonded state, likely to be one that had been relieved from the previously H-bonded state. Such dissociation of bonded carbonyl and simultaneously the occurrence of unassociated carbonyl were reported for the previously reported ABZ salts with several carboxylic acids, including MA,^21^ where the authors attributed it to blue-shifting of the ABZ ester C=O stretching from 1713 cm^–1^. In this work, with a cross-examination with MA C=O band shifts, however, we propose that at 1741 cm^–1^, it should be indicative of MA carboxylic acid C=O stretching (1704 cm^–1^) which had dissociated from its previous H-bonded dimeric structure, with a counterpart C=O from the same dissociation appearing at a far lower wavenumber (1513 cm^–1^) in the case of the equimolar salt, owing to protonation,^35^ or slightly red-shifted to give a broad band with a peak at 1696 cm^–1^ in the 1:2 eutectic due to stronger interspecies intermolecular H-bonding with the secondary N–H functions (from both the imidazole ring and the amide) within ABZ. The medium peak representing the ABZ ester C=O stretching at 1713 cm^–1^, on the other hand, was more likely to have red-shifted to 1702 cm^–1^ in the equimolar salt possibly following change from same-species intermolecular H-bonding to intramolecular H-bonding and blue-shifted to 1719 cm^–1^ in the 1:2 eutectic presumably after H-bonding with the carboxylic acid C=O from MA. The blue-shift was likely due to the fact that the interspecies intermolecular H-bonding between ABZ and MA would have been stronger than the intramolecular ABZ C=O–H–N H-bond since the oxygen atom would provide greater electronegativity compared to the nitrogen atom.^36^

Accompanying the above-mentioned band shifts, the intramolecularly H-bonded carbonyl doublet with peaks at 1620 and 1631 cm^–1^ was also seen to have become one significantly blue-shifted broad peak at 1657 cm^–1^ in the equimolar salt implying potential loss of the tautomeric copresence while showing mild blue-shifts to slightly higher wavenumbers (1624 and 1637 cm^–1^, respectively) in the 1:2 eutectic, suggesting weakened strengths of the interactions that these carbonyl groups are involved in.

In the N–H region, ABZ’s carbamate N–H stretching was seen to have red-shifted from 3320 to 3188 cm^–1^ in both the salt and the eutectic products, suggesting that they were involved in stronger H-bonding networking, with the MA carbonyl, following LAG.^37^

**Figure 7:**
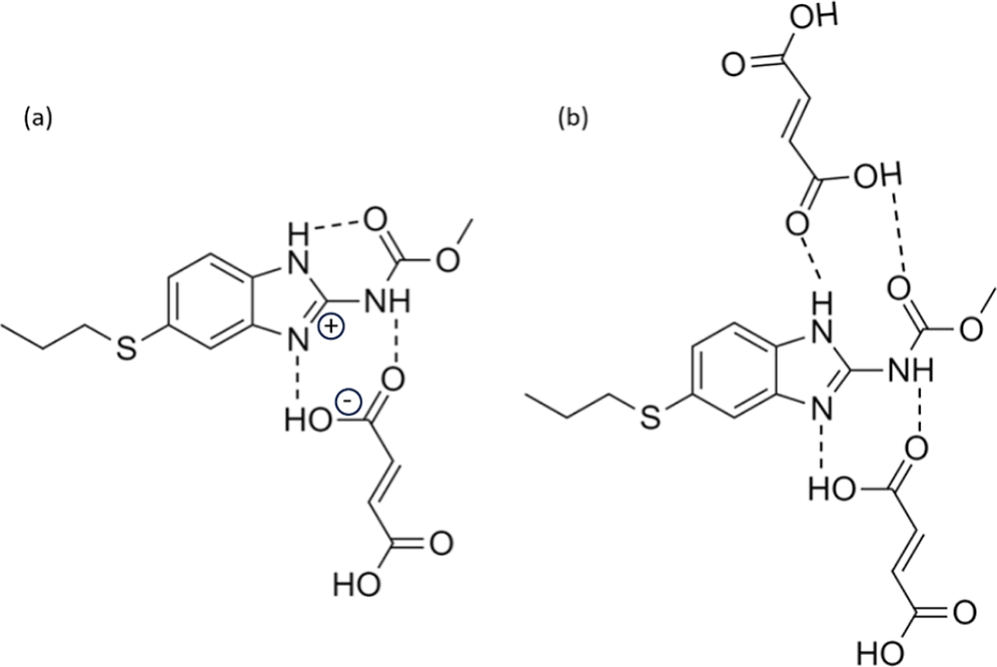
Proposed interaction networks between ABZ and MA within (a) χABZ = 0.50 salt and (b) χABZ = 0.33 eutectic. The suggested structure for the equimolar salt was hypothesized based on the proposed ionic synthon between ABZ and carboxylic acid in an *R*_2_^2^ (8) motif in the literature^38^ and adapted to reflect the said synthon between an ABZ–MA pair.

A summarized band assignment is presented in Table 2. Based on the above interpretation of the FTIR results, the following structures can be proposed for the ABZ–MA χABZ = 0.50 salt and χABZ = 0.33 eutectic (Figure 7). Due to the difficulties in finding an appropriate solvent to grow crystals of a suitable dimension, single-crystal elucidation was not able to be carried out for the ABZ–MA salt or eutectic within the scope of this work. Therefore, these structures remain as suggestions based on the data gathered.

**Table 2:**
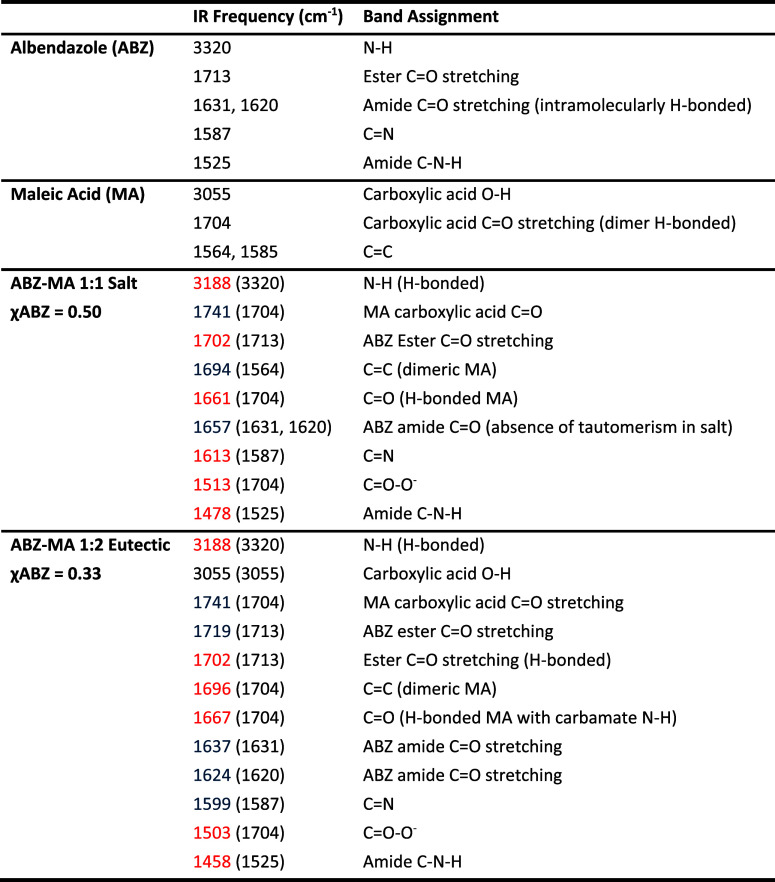
FTIR Assignment of the Main Absorption Bands for ABZ, MA, the Equimolar ABZ–MA Salt, and the 1:2 ABZ–MA Eutectic^a^

a Note the values presented in the parentheses represent the original band positions in the pure parent compounds, whereas the colors represent whether the band was observed to have red- or blue-shifted.

### Quantification of the ABZ–MA Eutectic within a Polymeric Matrix

The complexity of the ABZ–MA systems in the characteristic FTIR band regions has been demonstrated in the previous section. With the hypothesized in situ eutectic synthesis and concurrent formulation using HMRE in mind, it was thought to be extremely challenging to identify a single peak or a small number of unique peaks to enable quantification of the ABZ–MA 1:2 eutectic that could be present within a polymeric matrix. To achieve this, a multivariate analysis calibration model was developed by combining vibrational spectroscopy with chemometric to resolve overlapping of spectral information and to magnify minor differences.^39−43^

PLS regression, as an extension of principal component analysis (PCA), is often used to connect the information between primary variables (*X*) and response variables (*Y*), using a linear multivariate model, with the goal to be able to accurately predict *Y* from *X*.^26^ A PLS model is advantageous over other calibration tools, such as multiple linear regression (MLR), when working with spectroscopic data, since a PLS model is capable of analyzing data with strongly correlated variables, a task that the likes of MLR cannot perform.^44^

A preliminary model was constructed during the early stage of this study, using a series of ternary mixtures containing varying ratios of the ABZ–MA 1:2 eutectic with a physical mixture of ABZ and MA blended at a 1:2 molar ratio,^45^ in an attempt to explore the feasibility of quantitatively determining eutectic formation. In the preliminary model, the entire midrange IR spectrum (650–4000 cm^–1^) was used, allowing subtle band transitions to be included within the model, with the aim to produce a calibration curve with greater accuracy.^43^ The preliminary model utilizing the first derivative filter, a commonly used preprocessing filter to reduce scattering effects, resulted in the greatest accuracy and predictability (data available in the Supporting Information). The score plot using the first derivative filtered spectra showed that most scores remained within the Hotelling’s *T*^2^ ellipse (data shown in Supporting Information). The PLS model developed contained two PC, with PC1 accounting for most of the variation (83.2%).

With the presence of a polymeric excipient, the preliminary model was modified based on the ABZ–MA–EPO formulation, assuming that eutectic formation within the EPO matrix would not be accompanied by amorphization of ABZ or MA for practical simplicity. In addition, the IR spectral pattern of EPO was compared to that of the eutectic in order to determine regions of the spectra where the minimum polymer interference to the detection of the eutectic was present. These regions were determined to be present between 3035–3265, 1500–1675, and 650–735 cm^–1^ (Supporting Information Figure 3).

Interestingly, with the presence of EPO and the use of the above identified specific regions, this latter model found the greatest accuracy and predictability after the application of the second derivative filter (Supporting Information). It has been reported in the literature that the application of the second derivative filter could be advantageous, where not just minor differences in spectra need to be enhanced, but noticeable baseline drifts need to be eradicated.^46^ Score plot following application of the first derivative filter identified several outliers from the physical mixture of ABZ and MA (where 0% eutectic was expected) (Supporting Information Figures 4a and 5). After removal of these outliers, and the application of the second derivative filter, the score plot showed only one outlier outside of the Hotelling’s *T*^2^ ellipse, with component 1 (PC1) accounting for 84.7% of the variation, while the overall model accounts for 89.4% of the variation within the data set (Supporting Information Figure 4b).

**Figure 8:**
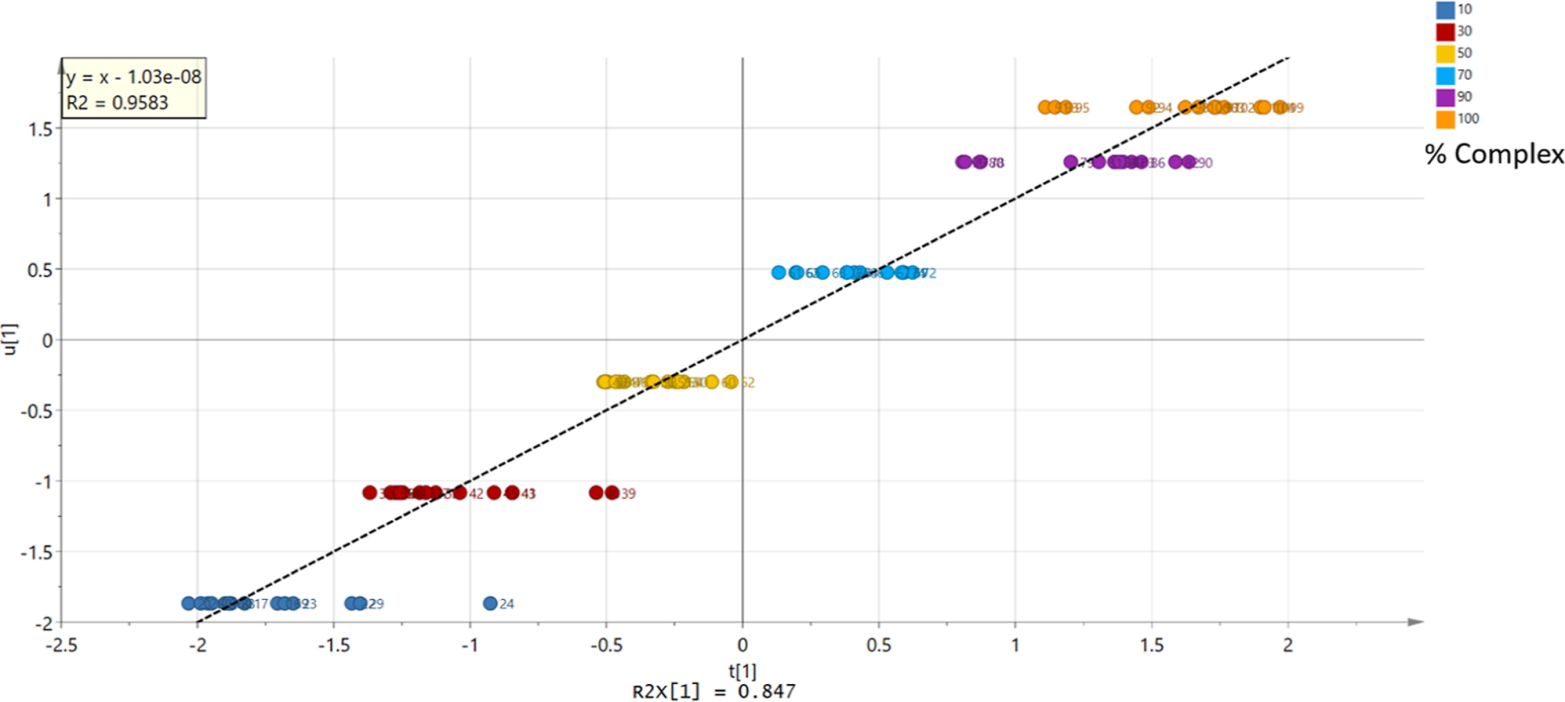
Plot showing *t*_1_ vs *u*_1_ for data after application of the second derivative filter and removal of outliers (*R*^2^ = 0.9583). The score plot was colored according to the percentage of the ABZ–MA 1:2 eutectic.

A positive linear correlation was observed from the scatterplot of the PC1 score vectors (*u*_1_ vs *t*_1_), with a good linearity (*R*^2^ > 0.96) obtained using the second derivative filtered data (Figure 8).

### Validation of the PLS Calibration Model

Following successful development, the eutectic quantification PLS model was validated for accuracy/precision using samples that contained the eutectic at concentrations that were not included in the calibration model.

**Figure 9:**
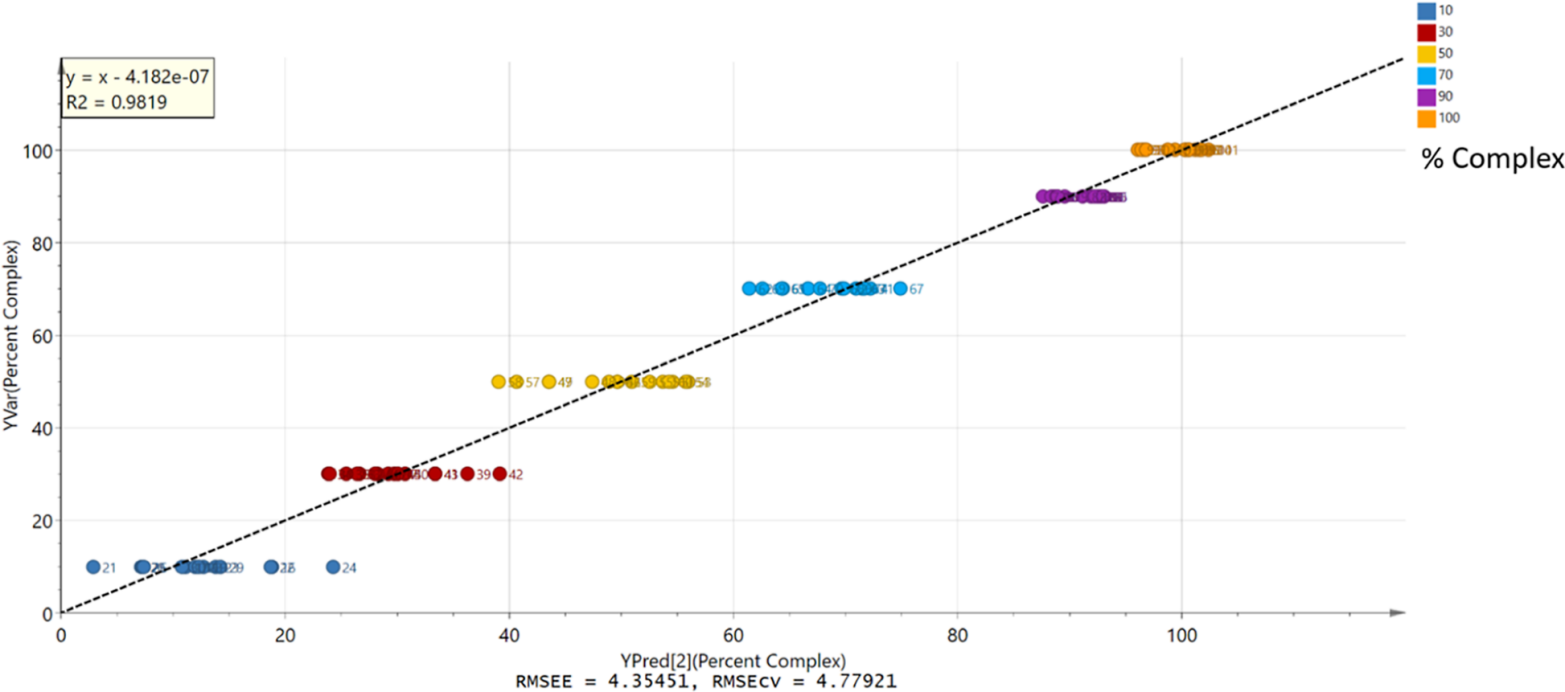
Observed vs predicted plot for the filtered model, showing an error of prediction of 4.78%. The plot was colored according to the percentage of the ABZ–MA 1:2 eutectic.

The observed-versus-predicted plot (Figure 9) demonstrated a good linearity with an *R*^2^ value of 0.982 and accuracy with an RMSEcv of 4.78%, which lies well within the recommended range of 10.00%.^39^ Response permutation was also carried out in order to provide an indication of the statistical significance of the estimated predictive power of the calibration model. The response permutation plot (Supporting Information Figure 6) demonstrated that the model was valid, as the *Q*^2^ intercept is −0.151 and the *R*^2^ intercept is −0.005, values which must not exceed 0.05 and 0.4, respectively.^39^

External validation was also carried out using a series of four samples of known concentration of the eutectic, which were not used in model development (20, 40, 60, and 80%). Table 3 shows that the PLS model exhibited an excellent accuracy at high eutectic concentrations, with 100.8 ± 2.7% recovery of the theoretical eutectic content for the 80% validation sample. As the eutectic content decreases in the blend, so does the accuracy of the developed PLS model. This was not unexpected as the model was built on the grand assumption that the only compositional change during the process of eutectic formation within the EPO matrix was the formation of the ABZ–MA 1:2 eutectic and simultaneous reduction of the individual parent compound (ABZ and MA, respectively) contents. The assumption did not take into consideration the possibility of amorphization of the parent compound or the potential complexity of compound species within the final matrix. As a cationic polymer, EPO was hypothesized to not show substantial interactivity with the basic compound ABZ. However, the impact of p*K*_a_ differences between EPO and the other parent reagent, MA, and more importantly how the chemistry between ABZ and MA would be influenced by such are yet to be understood.

**Table 3:** Validation Data for the Predicted Eutectic Yield, for Four Different Concentrations of Eutectic

prepared eutectic content (%)	determined eutectic content (%)	recovery (%)
20	16.32 ± 7.96	81.6 ± 39.8
40	41.35 ± 5.86	103.4 ± 14.6
60	58.73 ± 2.94	97.9 ± 4.9
80	80.63 ± 2.15	100.8 ± 2.7

### In Situ ABZ–MA Eutectic Synthesis and Concurrent Formulation Using Single-Step HMRE

It was of interest to our team to find scalable and industrially friendly preparation methods for the production of novel drug-enabled formulation systems. With previous success of using continuous, end-to-end and industrial-ready HMRE for a one-step production of pharmaceutical cocrystal suspensions,^8,9^ it was hypothesized that concurrent synthesis and formulation of the ABZ–MA 1:2 eutectic within a polymeric matrix might be achievable using the same preparation mean.

In the construction of the IR-based PLS model, it was assumed that ABZ was unlikely to form an amorphous dispersion within the EPO matrix based on simple chemistry that the reaction does not occur between two basic compounds. However, as a hydrophilic polymer, there was also possibility that the mere presence of EPO could provide enhancement to the dissolution of ABZ by improving wettability. In order to gain a more comprehensive understanding of the impact of ABZ dissolution within a polymeric matrix eutectic formation, an ABZ–EPO binary formulation was also extruded as a comparison reference.

Extrusions were performed with zone temperatures set to 120 °C and a screw speed maintained at 30 rpm throughout processing. The temperature setting was one typically used for EPO-based matrices, whereas a screw speed at 30 rpm was chosen, as it was found to result in reasonably longer residence times, during preliminary trials, to best replicate the milling duration required for sufficient production of the eutectic in LAG in earlier work.

#### Extrusion of the ABZ–EPO Binary System

**Figure 10:**
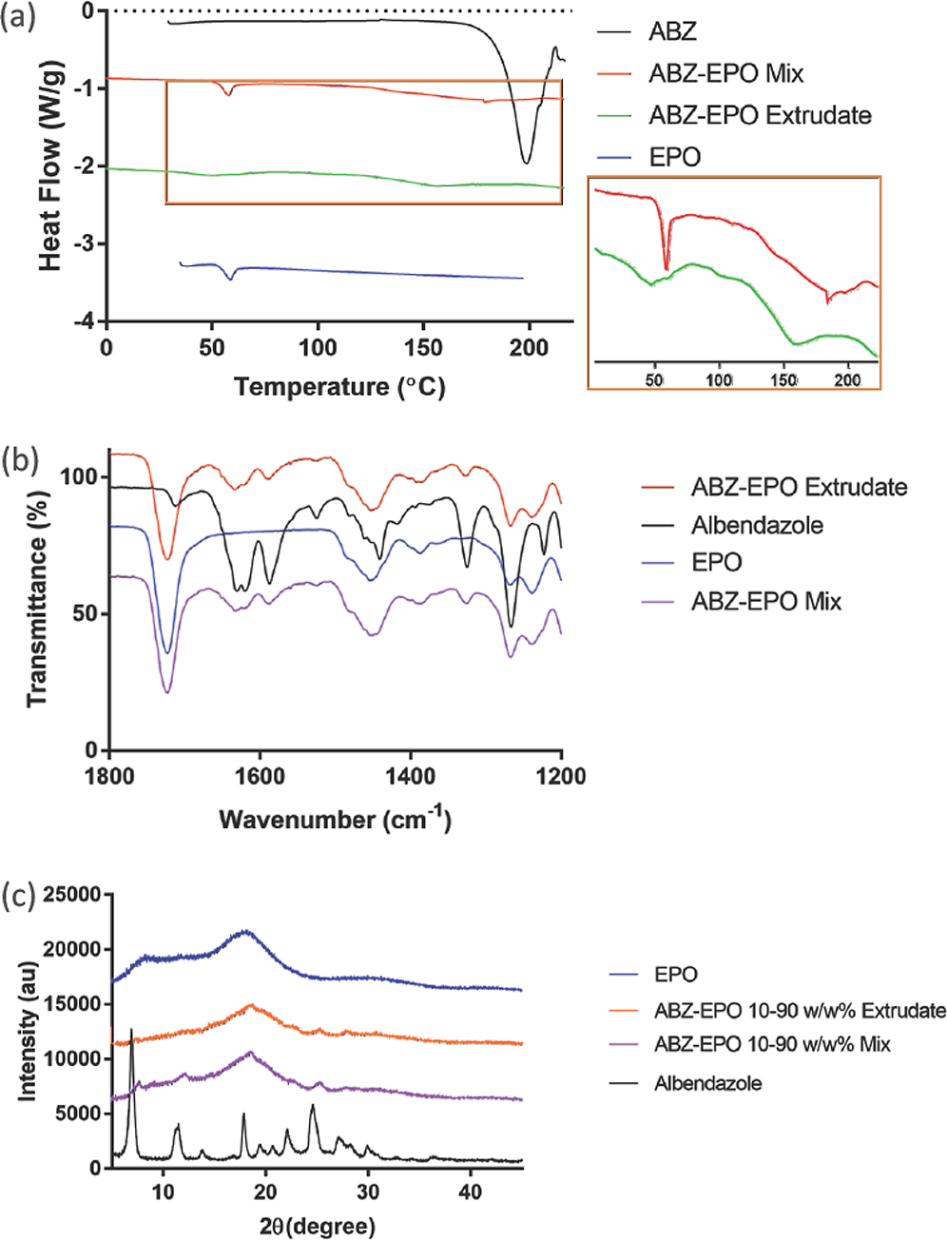
Overlain comparisons of the DSC thermograms (a), FTIR spectra (b), and PXRD patterns (c), respectively, comparing ABZ, EPO, physical mixture of ABZ and EPO at 10% w/w ABZ, and the corresponding extrudate.

EPO exhibited a glass transition event with a midpoint at 53.08 ± 1.38 °C, accompanied by a fronting endothermic relaxation, when analyzed on its own. In the physically mixed binary blend containing 10% ABZ, the EPO *T*_g_ was observed at 52.79 ± 0.52 °C, followed by a small and depressed *T*_m_ for ABZ with the peak measured at 181.30 ± 2.31 °C (Figure 10a). Such a melting peak depression should be attributed to the low ABZ loading and disruption of the ABZ entropy by the presence of a large amount of EPO and should not be mistakenly attributed to a loss of crystallinity in this mixture. This melting depression was observed to have reached an even greater extent following extrusion, where the onset of melting was found at 131.61 ± 2.05 °C, and the peak was found at 154.55 ± 0.42 °C, respectively. A broadened glass transition was also observed for EPO where the midpoint had depressed to 38.49 ± 0.14 °C in the extrudate. Although the catalysts exhibited concurrent thermal event depression, there was no evidence, in the thermograms, that ABZ and EPO could have formed a miscible system to any extent.

The same conclusion might be drawn based on the FTIR spectra where no pronounced band shift or unique absorption band was observed in either the physical mixture or the extruded ABZ–EPO binaries, which was an indication that there were minimal interactions, if not none whatsoever, occurring between ABZ and EPO during the extrusion (Figure 10b).

A comparison of the PXRD patterns before and after extrusion was also able to indicate that a substantial portion of the loaded ABZ remained crystalline and undisrupted of the original crystal structure (Figure 10c), again suggesting very little interaction occurring between ABZ and EPO, during the extrusion process.

#### Reactive Extrusion of the ABZ–MA–EPO Ternary System

**Figure 11:**
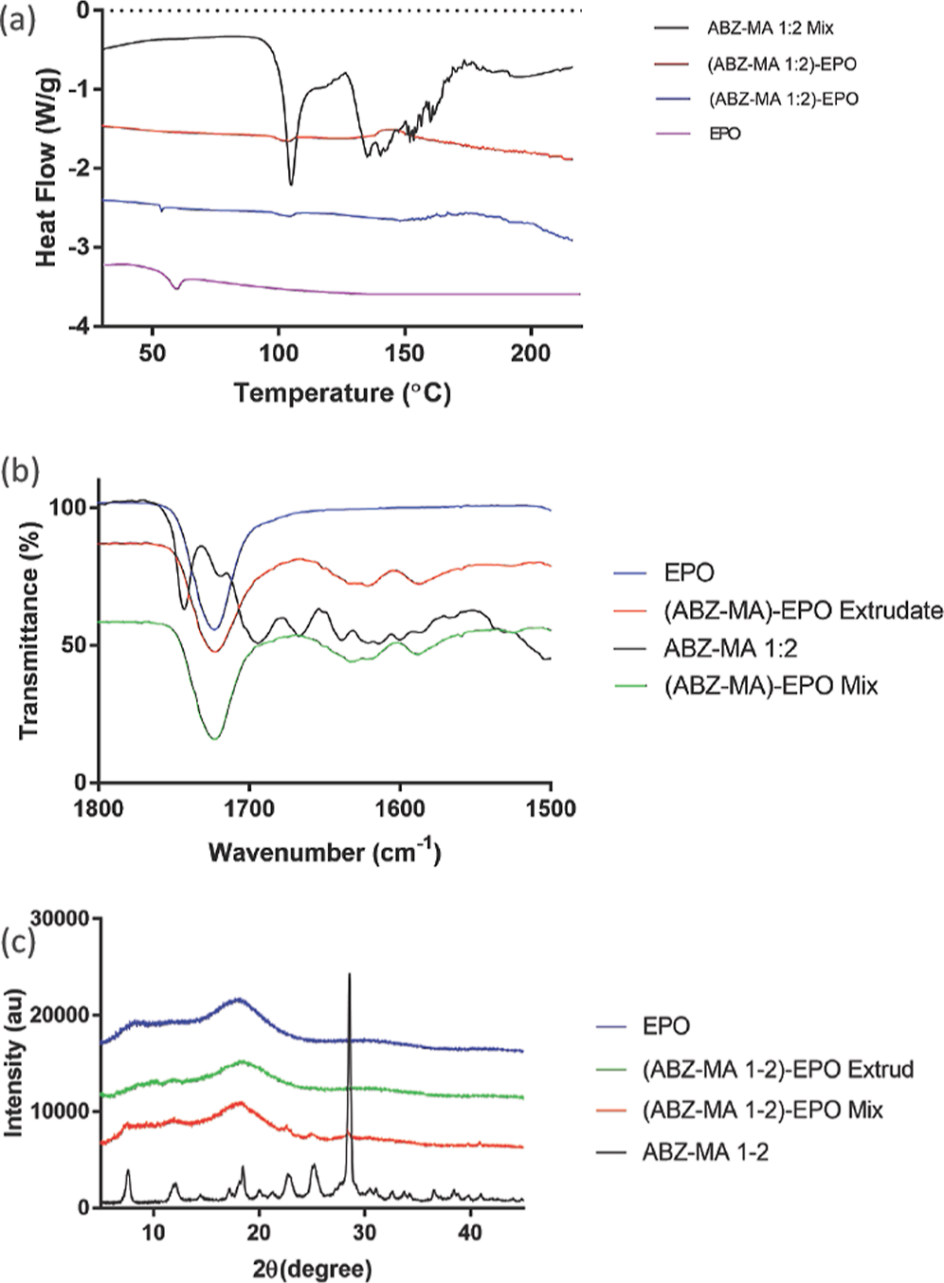
Overlain comparisons of the DSC thermograms (a), FTIR spectra (b), and PXRD patterns (c), respectively, comparing ABZ–MA 1:2 eutectic, EPO, physical mixture of the ABZ–MA eutectic and EPO at a drug loading equivalent to 10% w/w ABZ, and the corresponding extrudate.

Similar to the ABZ–EPO binary system, with the ABZ–MA–EPO ternary blend, the EPO *T*_g_ was also seen to have shifted to a lower temperature with the midpoint of the step-event measured at 49.07 ± 5.67 °C in the extrudate (Figure 11a). An ABZ–MA physical mixture at 1:2 molar ratio was examined, as opposed to using just ABZ, as a comparison reference, where formation of the eutectic adduct was observed at 100.94 ± 1.17 °C. The melting for MA and ABZ in their original forms, though, was difficult to identify with a plethora of cramped up small thermal events possibly as a result of thermal instability from approximately 130 °C and onward.

In both the ternary physical mixture and extrudate, interestingly, a new endothermic event was observed at 98.03 ± 0.44 and 95.88 ± 1.39 °C, respectively, suggesting a lightly depressed melting of the ABZ–MA 1:2 eutectic. The occurrence of the eutectic’s melting in the DSC thermogram for the ternary physical mixture was a positive sign and testament of the strong reactivity and interaction specificity between the eutectic parent reagents, ABZ and MA. It was evident that not only the eutectic formation could be triggered with mere elevation of the temperature, but it also takes priority even when the reagents were “diluted” by a substantial amount of a polymeric excipient.

The FTIR spectra, however, were not able to provide a clear sign of the eutectic presence, possibly owing to the low drug loading within the system, as well as the subtlety in the eutectic-unique band shifts (Figure 11b). In the PXRD patterns, however, it was obvious that loaded ABZ and MA had both lost their original crystallinity after extrusion (Figure 11c). Surprisingly, it was not possible to identify any characteristic peaks for the ABZ–MA 1:2 eutectic, which was evidently present in the DSC thermograms, in the extrudates. This being said, it is important that we keep in mind that the drug loading was kept reasonably low and hence there was possibly a significant dilution effect due to the presence of EPO. In addition, the eutectic from LAG was seen to exhibit substantially reduced peak intensities, when compared with the original crystalline ABZ and MA, which were likely produced using solvent-based synthesis and purification.

#### Determination of the Eutectic Yield Following ABZ–MA–EPO Ternary Extrusion

Using the developed multivariate PLS model, it was possible to quantify the amount of the ABZ–MA eutectic in the solid state after formulation in EPO using FTIR spectroscopy. This model was applied to spectroscopic data obtained after extruding a ternary physical mixture of ABZ, MA, and EPO under various conditions and using different extruders (Table 4). It would be interesting to note that with various shear forces and residence times within the extruder barrel, by altering the variety of conveying and kneading elements as well as the barrel length, surprisingly the processes always resulted in somewhat similar yields of the eutectic from the loaded amount of ABZ and MA. With this being said, though, it could also be seen that with the HAAKE Minilab, where the barrel was of a shorter length and very limited mixing was available, there was a greater variation of the eutectic yield, suggesting greater variation in the reaction kinetics and hence suboptimal quality control.

**Table 4:** Percentage Yield (%) of the ABZ–MA Eutectic Obtained after HME with EPO Using Different Extruders and Employing Different Screw Elements

extruder	eutectic yield (%)
MiniLab—nonintermeshing corotating TSE	81.30 ± 6.61
Rondol—intermeshing corotating TSE employing only FC elements	86.82 ± 1.33
Rondol—intermeshing corotating TSE employing FC and MIX elements	87.97 ± 1.09

These observations would have suggested the possibility of a negative effect, again caused by the low drug loading within the matrices. With a low drug loading, the spacing between near particles from both parent reagent species would have been more significant, and hence, distribution of the eutectic yield was more influenced by the mixing intensity within the extruder barrel and the residence time of the whole process.

It is also important to point out that the potential interactivity between the cationic polymer EPO and the acidic compound MA has not been studied in this work. Although the eutectic formation took preference in the ABZ–MA–EPO ternary blend, a small portion of competitive interaction of some sort could have led to incomplete eutectic yielding.

### In Vitro Drug Dissolution

**Figure 12:**
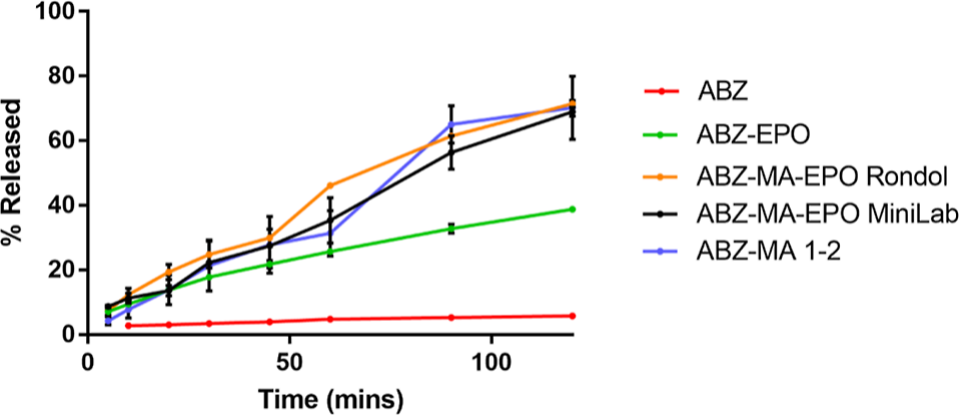
Cumulative percentage of ABZ released over a 2 h period, using various systems and/or formulations containing equivalent to 200 mg of ABZ, in a 900 mL blank simulated gastric fluid (pH 1.2).

A comparison of the dissolution profiles between dispersions of ABZ and ABZ–MA 1:2 in EPO is shown in Figure 12. EPO is water-soluble (at the pH of the tested media) and will promote the wettability and potentially the rate of dissolution for ABZ, causing ABZ–MA–polymer formulations to possibly exhibit a synergistic effect between both the water-soluble polymer and coformer. Each extrudate contained the same weight equivalent of ABZ (10% w/w). When ABZ was extruded with EPO as a binary matrix, the maximum drug release observed after 2 h was 38.8 ± 0.9%, showing an almost 7-fold increase compared to pure ABZ (5.75 ± 0.53%). The dissolution profile for this binary extrudate was largely linear (*R*^2^ > 0.98 using a simple linear regression fit), suggesting a dominant effect of matrix erosion on the drug release and dissolution throughout the 2 h time frame.

When mixtures of ABZ and MA at a 1:2 ratio were extruded with EPO, at equivalent to 10% w/w ABZ loading in the final formulation, a significantly further enhanced dissolution behavior was recorded, when compared to the ABZ–EPO binary matrix, with the ternary formulations exhibiting doubled extent and rate of dissolution than the binary system (Figure 12). Notably, the dissolution profiles of the ABZ–MA–EPO system extruded using the mildly mixing Minilab and the significantly increased mixing Rondol were almost identical, in terms of both the extent and rate of dissolution, at the majority of time points throughout the experiments (Supporting Information). This correlated well with the statistically similar eutectic yields between extrusions performed using different equipment and incorporating various screw profiles, determined in the previous section. Such results would suggest that the incomplete parent reagents-to-eutectic conversion was due to saturation of eutectic formation between available parent reagents and that a potentially competitive interaction pattern was present in the ternary mixture.

The highest drug percentages recorded throughout the time frame of the experiments occurred at the 2 h time point at 68.9 ± 1.3% for the Minilab extrudates and 71.5 ± 1.0% for the Rondol extrudates, respectively. Both values were below the expected eutectic content. However, it should be noted that the dissolution profiles did not reach a plateau or showing noticeable sign of the rate of dissolution to slow down at the 2 h point. Should the dissolution experiments have carried on, it was likely that the final extent of dissolution would have reached the expected values.

## Conclusion

A novel ABZ–MA eutectic system has been identified and successfully prepared in isolation using mechanochemical preparations. This eutectic was characterized using a variety of solid-state characterization techniques, and it was found to offer enhanced dissolution compared to the original drug compound. It was, therefore, postulated that an enhanced dissolution rate, solubility, and potential bioavailability of ABZ could be achieved through the formation of this eutectic version of the drug, embedded within a hydrophilic matrix.

The intermolecular interactions within the eutectic were confirmed by using spectroscopic analysis and were used to build a multivariate PLS calibration model to determine the yield of eutectic formation from a ternary mixture of the raw parent reagents and a polymer. Evaluation of the influence of eutectic formation on the release of ABZ was established by manufacturing extrudates containing only the pure drug with the polymeric excipient. Good reactivity and reaction selectivity were confirmed between ABZ and MA in the presence of the investigated polymer, EPO. The EPO-based matrices extruded using a binary ABZ–EPO blend and that using a ternary ABZ–MA–EPO blend both resulted in greatly enhanced dissolution performances when compared with pure ABZ.

By demonstrating the usefulness of the established multivariate PLS calibration model in determining the reaction yield for in situ eutectic formation in a polymeric matrix, future work may potentially proceed to convert the offline yield determination to an online or in-line analysis in a timely manner. In so doing, it may enable real-time monitoring and control of the process and product quality during HMRE preparations involving in situ reaction and concurrent formulation, demonstrating a good alignment with the ongoing industrial interest in end-to-end production and continuous manufacturing.

## Supporting Information Available

Overlain of representative DSC thermograms for binary mixtures of ABZ and MA, at varying mole fractions of ABZ, following LAG; percentage yield of the 1:2 ABZ–MA eutectic during binary LAG, calculated using a PLS model after grinding for varying time periods at (a) 20, (b) 22, and (c) 30 Hz, respectively; FTIR spectral regions, 3035–3265, 1500–1675, and 650–735 cm^–1^, where EPO was found to show negligible interference to the formation of the ABZ–MA 1:2 eutectic; calculated parameters from several versions of the PLS model when data were preprocessed using different spectral filters; score plot of data after application of the first (a) and second (b) derivative filters, respectively, (*t*_1_ vs *t*_2_), containing two PCs; Hotelling’s *T*^2^ plot, showing three suspect outliers and no major outliers in the data after application of the second derivative filter; response permutation plot for validation of the developed PLS model; validation of the developed PLS model using observed versus predicted eutectic content (%); and drug dissolution kinetics of the EPO extrudates containing ABZ and ABZ–MA 1:2, respectively (PDF)

## Notes

Published as part of *Molecular Pharmaceutics*special issue “Advances in Small and Large Molecule Pharmaceutics Research across Ireland”.

## References

[ref1] Berry D. J.; Steed J. W. Pharmaceutical Cocrystals, Salts and Multicomponent Systems; Intermolecular Interactions and Property Based Design. Adv. Drug Delivery Rev. 2017, 117, 3–24. 10.1016/j.addr.2017.03.003. 28344021

[ref2] Sarma B.; Chen J.; Hsi H. Y.; Myerson A. S. Solid Forms of Pharmaceuticals: Polymorphs, Salts and Cocrystals. Korean J. Chem. Eng. 2011, 28 (2), 315–322. 10.1007/s11814-010-0520-0.

[ref3] Stoler E.; Warner J. C. Non-Covalent Derivatives: Cocrystals and Eutectics. Molecules 2015, 20 (8), 14833–14848. 10.3390/molecules200814833. 26287141 PMC6332263

[ref4] Cooke C. L.; Davey R. J.; Black S.; Muryn C.; Pritchard R. G. Binary and Ternary Phase Diagrams as Routes to Salt Discovery: Ephedrine and Pimelic Acid. Cryst. Growth Des. 2010, 10 (12), 5270–5278. 10.1021/cg1011296.

[ref5] Fucke K.; Myz S. A.; Shakhtshneider T. P.; Boldyreva E. V.; Griesser U. J. How Good Are the Crystallisation Methods for Co-Crystals? A Comparative Study of Piroxicam. New J. Chem. 2012, 36 (10), 1969–1977. 10.1039/c2nj40093f.

[ref6] Lee H. L.; Vasoya J. M.; Cirqueira M. d. L.; Yeh K. L.; Lee T.; Serajuddin A. T. M. Continuous Preparation of 1:1 Haloperidol-Maleic Acid Salt by a Novel Solvent-Free Method Using a Twin Screw Melt Extruder. Mol. Pharm. 2017, 14 (4), 1278–1291. 10.1021/acs.molpharmaceut.7b00003. 28245127

[ref7] Kasten G.; Nouri K.; Grohganz H.; Rades T.; Löbmann K. Performance Comparison between Crystalline and Co-Amorphous Salts of Indomethacin-Lysine. Int. J. Pharm. 2017, 533 (1), 138–144. 10.1016/j.ijpharm.2017.09.063. 28947246

[ref8] Li S.; Yu T.; Tian Y.; McCoy C. P.; Jones D. S.; Andrews G. P. Mechanochemical Synthesis of Pharmaceutical Cocrystal Suspensions via Hot Melt Extrusion: Feasibility Studies and Physicochemical Characterization. Mol. Pharm. 2016, 13, 3054–3068. 10.1021/acs.molpharmaceut.6b00134. 27314248

[ref9] Li S.; Yu T.; Tian Y.; Lagan C.; Jones D. S.; Andrews G. P. Mechanochemical Synthesis of Pharmaceutical Cocrystal Suspensions via Hot Melt Extrusion: Enhancing Cocrystal Yield. Mol. Pharm. 2018, 15 (9), 3741–3754. 10.1021/acs.molpharmaceut.7b00979. 29166563

[ref10] Shan N.; Toda F.; Jones W. Mechanochemistry and co-crystal formation: effect of solvent on reaction kineticsElectronic supplementary information (ESI) available for PXRD profiles showing the grinding results for CTA + Bipy with and without solvent as well as CTA + 2fPh with different solvents. Chem. Commun. 2002, 2 (20), 2372–2373. 10.1039/b207369m. 12430446

[ref11] Hasa D.; Carlino E.; Jones W. Polymer-Assisted Grinding, a Versatile Method for Polymorph Control of Cocrystallization. Cryst. Growth Des. 2016, 16, 1772–1779. 10.1021/acs.cgd.6b00084.

[ref12] Thakur A.; Thipparaboina R.; Kumar D.; Sai Gouthami K.; Shastri N. R. Crystal Engineered Albendazole with Improved Dissolution and Material Attributes. CrystEngComm 2016, 18 (9), 1489–1494. 10.1039/C5CE02306H.

[ref13] Jung H.; Medina L.; García L.; Fuentes I.; Moreno-Esparza R. Absorption Studies of Albendazole and Some Physicochemical Properties of the Drug and Its Metabolite Albendazole Sulphoxide. J. Pharm. Pharmacol. 2011, 50 (1), 43–48. 10.1111/j.2042-7158.1998.tb03303.x. 9504433

[ref14] Noorani L.; Stenzel M.; Liang R.; Pourgholami M. H.; Morris D. L. Albumin Nanoparticles Increase the Anticancer Efficacy of Albendazole in Ovarian Cancer Xenograft Model. J. Nanobiotechnol. 2015, 13 (1), 25 10.1186/s12951-015-0082-8. PMC4409778 25890381

[ref15] Pourgholami M. H.; Akhter J.; Wang L.; Lu Y.; Morris D. L. Antitumor Activity of Albendazole against the Human Colorectal Cancer Cell Line HT-29: In Vitro and in a Xenograft Model of Peritoneal Carcinomatosis. Cancer Chemother. Pharmacol. 2005, 55 (5), 425–432. 10.1007/s00280-004-0927-6. 15565325

[ref16] Jiménez de los Santos C. J.; Pérez-Martínez J. I.; Gómez-Pantoja M. E.; Moyano J. R. Enhancement of Albendazole Dissolution Properties Using Solid Dispersions with Gelucire 50/13 and PEG 15000. J. Drug Deliv. Sci. Technol. 2017, 42, 261–272. 10.1016/j.jddst.2017.03.030.

[ref17] Kalaiselvan R.; Mohanta G. P.; Manna P. K.; Manavalan R. Inhibition of Albendazole Crystallization in Poly(Vinylpyrrolidone) Solid Molecular Dispersions. Pharmazie 2006, 61 (7), 618–624. 16889070

[ref18] Torrado S.; Torrado S.; Torrado J. J.; Cadórniga R. Preparation, Dissolution and Characterization of Albendazole Solid Dispersions. Int. J. Pharm. 1996, 140 (2), 247–250. 10.1016/0378-5173(96)04586-3.

[ref19] Hengsawas Surasarang S.; Keen J. M.; Huang S.; Zhang F.; McGinity J. W.; Williams R. O. Hot Melt Extrusion versus Spray Drying: Hot Melt Extrusion Degrades Albendazole. Drug Dev. Ind. Pharm. 2017, 43 (5), 797–811. 10.1080/03639045.2016.1220577. 27616147

[ref20] Priotti J.; Baglioni M. V.; García A.; Rico M. J.; Leonardi D.; Lamas M. C.; Menacho Márquez M. Repositioning of Anti-Parasitic Drugs in Cyclodextrin Inclusion Complexes for Treatment of Triple-Negative Breast Cancer. AAPS PharmSciTech 2018, 19 (8), 3734–3741. 10.1208/s12249-018-1169-y. 30255471

[ref21] Bolla G.; Nangia A. Novel Pharmaceutical Salts of Albendazole. CrystEngComm 2018, 20 (41), 6394–6405. 10.1039/C8CE01311J.

[ref22] Paulekuhn G. S.; Dressman J. B.; Saal C. Salt Screening and Characterization for Poorly Soluble, Weak Basic Compounds: Case Study Albendazole. Pharmazie 2013, 68 (7), 555–564. 10.1691/ph.2013.6507. 23923637

[ref23] Chattah A. K.; Zhang R.; Mroue K. H.; Pfund L. Y.; Longhi M. R.; Ramamoorthy A.; Garnero C. Investigating Albendazole Desmotropes by Solid-State NMR Spectroscopy. Mol. Pharm. 2015, 12 (3), 731–741. 10.1021/mp500539g. 25584993

[ref24] Yalkowsky S. H.; He Y.; Jain P. Handbook of Aqueous Solubility Data, 2nd ed.; CRC Press, Taylor & Francis Group, 2010.

[ref25] Nastaj A.; Wilczynski K. Optimization for Starve Fed/Flood Fed Single Screw Extrusion of Polymeric Materials. Polymers 2020, 12 (1), 149–217. 10.3390/polym12010149. 31936045 PMC7022554

[ref26] Wold S.; Sjöström M.; Eriksson L. PLS-Regression: A Basic Tool of Chemometrics. Chemom. Intell. Lab. Syst. 2001, 58 (2), 109–130. 10.1016/S0169-7439(01)00155-1.

[ref27] Long F. H. Multivariate Analysis for Metabolomics and Proteomics Data. In Proteomic and Metabolomic Approaches to Biomarker Discovery; Elsevier, 2013; pp 299–311.

[ref28] Lu E.; Rodríguez-Hornedo N.; Suryanarayanan R. A Rapid Thermal Method for Cocrystal Screening. CrystEngComm 2008, 10 (6), 665–668. 10.1039/b801713c.

[ref29] Cherukuvada S.; Nangia A. Eutectics as Improved Pharmaceutical Materials: Design, Properties and Characterization. Chem. Commun. 2014, 50 (8), 906–923. 10.1039/C3CC47521B. 24322207

[ref30] Chattah A. K.; Zhang R.; Mroue K. H.; Pfund L. Y.; Longhi M. R.; Ramamoorthy A.; Garnero C. Investigating Albendazole Desmotropes by Solid-State NMR Spectroscopy. Mol. Pharm. 2015, 12 (3), 731–741. 10.1021/mp500539g. 25584993

[ref31] Pranzo M. B.; Cruickshank D.; Coruzzi M.; Caira M. R.; Bettini R. Enantiotropically Related Albendazole Polymorphs. J. Pharm. Sci. 2010, 99 (9), 3731–3742. 10.1002/jps.22072. 20112428

[ref32] Hengsawas Surasarang S.; Keen J. M.; Huang S.; Zhang F.; McGinity J. W.; Williams R. O. Hot Melt Extrusion versus Spray Drying: Hot Melt Extrusion Degrades Albendazole. Drug Dev. Ind. Pharm. 2017, 43 (5), 797–811. 10.1080/03639045.2016.1220577. 27616147

[ref33] Stott P. W.; Williams A. C.; Barry B. W. Transdermal Delivery from Eutectic Systems: Enhanced Permeation of a Model Drug, Ibuprofen. J. Controlled Release 1998, 50 (1–3), 297–308. 10.1016/S0168-3659(97)00153-3. 9685897

[ref34] Nie B.; Stutzman J.; Xie A. A Vibrational Spectral Maker for Probing the Hydrogen-Bonding Status of Protonated Asp and Glu Residues. Biophys. J. 2005, 88 (4), 2833–2847. 10.1529/biophysj.104.047639. 15653739 PMC1305378

[ref35] Oomens J.; Steill J. D. Free Carboxylate Stretching Modes. J. Phys. Chem. A 2008, 112 (15), 3281–3283. 10.1021/jp801806e. 18363393

[ref36] Prasad K. D.; Cherukuvada S.; Devaraj Stephen L.; Guru Row T. N. Effect of Inductive Effect on the Formation of Cocrystals and Eutectics. CrystEngComm 2014, 16 (42), 9930–9938. 10.1039/C4CE01489H.

[ref37] De Araujo G. L. B.; Ferreira F. F.; Bernardes C. E. S.; Sato J. A. P.; Gil O. M.; De Faria D. L. A.; Loebenberg R.; Byrn S. R.; Ghisleni D. D. M.; Bou-Chacra N. A.; Pinto T. J. A.; Antonio S. G.; Ferraz H. G.; Zemlyanov D.; Gonçalves D. S.; Minas Da Piedade M. E. A New Thermodynamically Favored Flubendazole/Maleic Acid Binary Crystal Form: Structure, Energetics, and in Silico PBPK Model-Based Investigation. Cryst. Growth Des. 2018, 18 (4), 2377–2386. 10.1021/acs.cgd.7b01807.

[ref38] Bolla G.; Nangia A. Novel Pharmaceutical Salts of Albendazole. CrystEngComm 2018, 20 (41), 6394–6405. 10.1039/C8CE01311J.

[ref39] Eriksson L.; Byrne T.; Johansson E.; Trygg J.; Vikstrom C. Multi- and Megavariate Data Analysis Basic Principles and Applications; Umetrics Academy: Sweden, 2006.

[ref40] Schoonover J. R.; Marx R.; Zhang S. L.; Roductio I. Multivariate Curve Resolution in the Analysis of Vibrational Spectroscopy Data Files. Appl. Spectrosc. 2003, 57, 154A–170A. 10.1366/000370203321666461. 14658670

[ref41] Helmy R.; Zhou G. X.; Chen Y. W.; Crocker L.; Wang T.; Wenslow R. M.; Vailaya A. Characterization and Quantitation of Aprepitant Drug Substance Polymorphs by Attenuated Total Reflectance Fourier Transform Infrared Spectroscopy. Anal. Chem. 2003, 75 (3), 605–611. 10.1021/ac020538i. 12585491

[ref42] Soares F. L. F.; Carneiro R. L. Evaluation of Analytical Tools and Multivariate Methods for Quantification of Co-Former Crystals in Ibuprofen-Nicotinamide Co-Crystals. J. Pharm. Biomed. Anal. 2014, 89, 166–175. 10.1016/j.jpba.2013.11.005. 24291798

[ref43] De Luca M.; Oliverio F.; Ioele G.; Ragno G. Multivariate Calibration Techniques Applied to Derivative Spectroscopy Data for the Analysis of Pharmaceutical Mixtures. Chemom. Intell. Lab. Syst. 2009, 96 (1), 14–21. 10.1016/j.chemolab.2008.10.009.

[ref44] Cozzolino D.; Cynkar W. U.; Shah N.; Smith P. Multivariate Data Analysis Applied to Spectroscopy: Potential Application to Juice and Fruit Quality. Food Res. Int. 2011, 44 (7), 1888–1896. 10.1016/j.foodres.2011.01.041.

[ref45] Bica K.; Shamshina J.; Hough W. L.; MacFarlane D. R.; Rogers R. D. Liquid Forms of Pharmaceutical Co-Crystals: Exploring the Boundaries of Salt Formation. Chem. Commun. 2011, 47 (8), 2267–2269. 10.1039/C0CC04485G. 21161097

[ref46] Barmpalexis P.; Karagianni A.; Nikolakakis I.; Kachrimanis K. Artificial Neural Networks (ANNs) and Partial Least Squares (PLS) Regression in the Quantitative Analysis of Cocrystal Formulations by Raman and ATR-FTIR Spectroscopy. J. Pharm. Biomed. Anal. 2018, 158, 214–224. 10.1016/j.jpba.2018.06.004. 29886369

