# Supplementary material for: In Situ Eutectic Formation in a Polymeric Matrix via Hot-Melt Reactive Extrusion and the Use of Partial Least Squares Regression Modeling for Reaction Yield Determination
Source: Mol Pharm. 2024 Aug 13;21(9):4272–84. doi: 10.1021/acs.molpharmaceut.4c00152 (PMC11372829; doi:10.1021/acs.molpharmaceut.4c00152)
Supplement: Supplementary file 1 — mp4c00152_si_001.pdf [file mp4c00152_si_001.pdf]

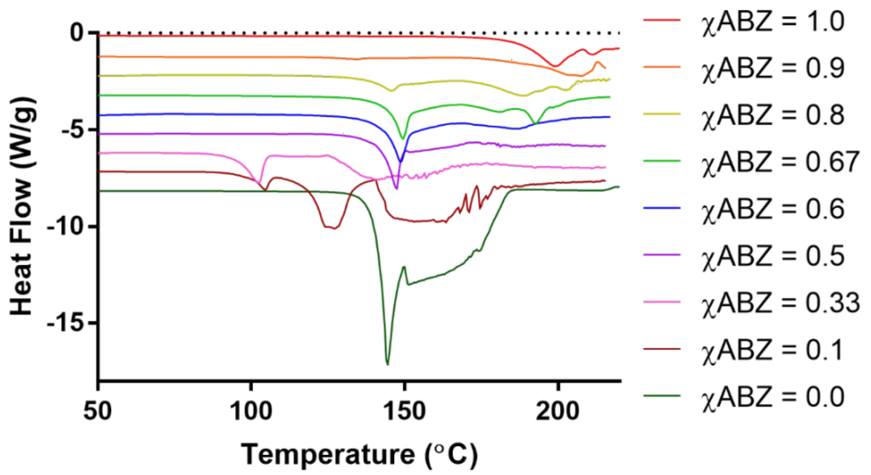

Supplementary Figure 1 Representative overlain DSC thermograms for binary mixtures of ABZ and MA after LAG at varying mole fractions of ABZ.

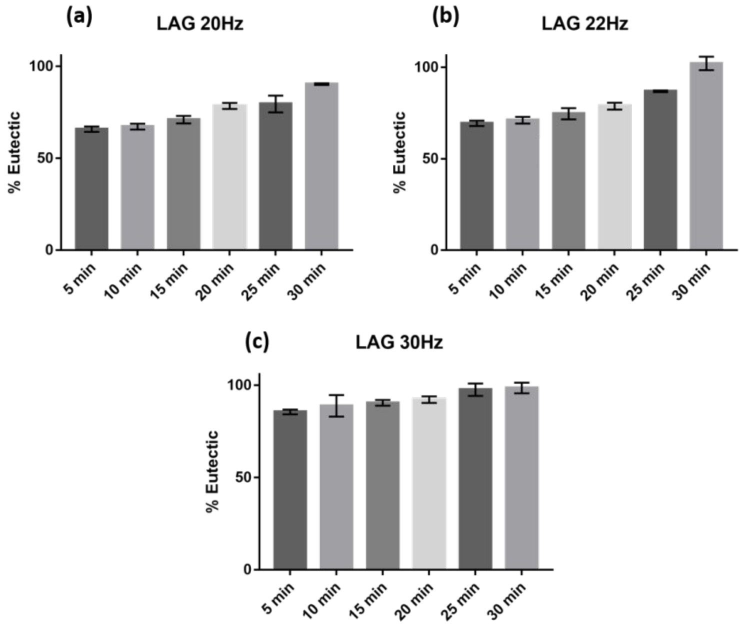

Supplementary Figure 2 Percentage yield of the 1:2 ABZ-MA eutectic during binary LAG, calculated using a PLS model after grinding for varying time periods at (a) 20 Hz, (b) 22 Hz and (c) 30 Hz, respectively.

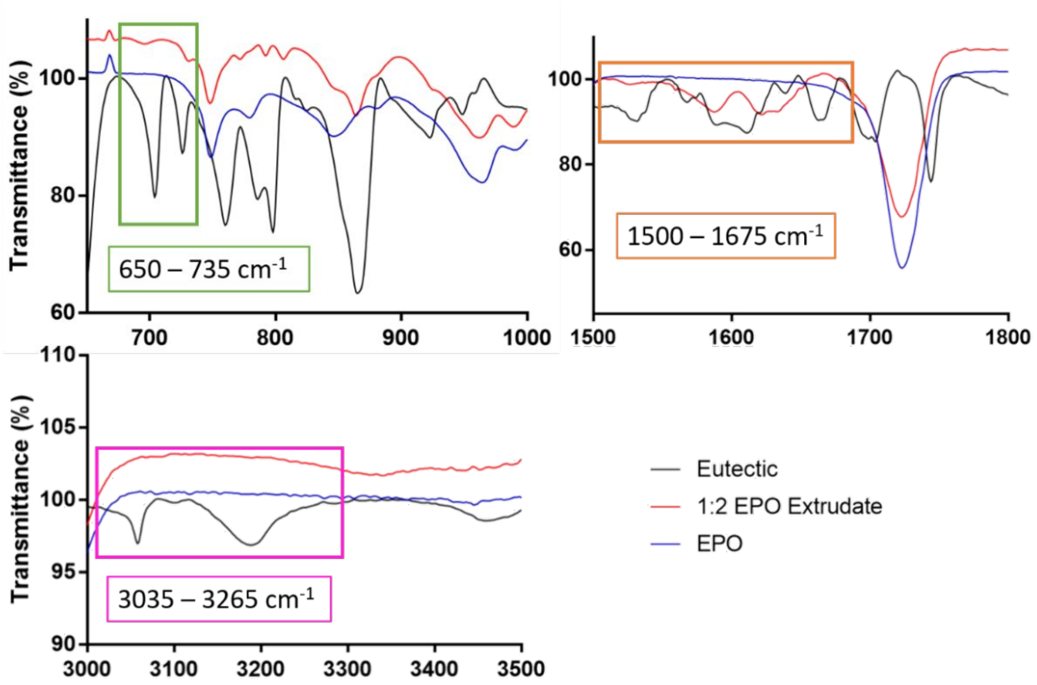

Supplementary Figure 3 FT-IR spectral regions, 3035 - 3265 cm^{-1}, 1500 - 1675 cm^{-1}, and 650 - 735 cm^{-1}, where EPO was found to show negligible interference to the formation of the ABZ-MA 1:2 eutectic.

Supplementary Table 1 Calculated parameters from several versions of the PLS model when data were pre-processed using different spectral filters.*

Spectral Filter	R^2X	R^2Y	Q^2	RMSEE	RMSEcv
Raw data	0.963	0.944	0.934	8.62	9.20
SNV	0.945	0.976	0.974	5.70	5.85
MSC	0.950	0.974	0.972	5.89	6.05
1st Derivative	0.958	0.978	0.974	5.45	6.07
2nd Derivative	0.957	0.980	0.977	5.19	5.58
Savitzky-Golay	0.963	0.946	0.935	8.53	9.15

** R^2X, R^2 predicted; R^2Y, R^2 observed; Q^2, indication of model validity; RMSEE, root mean square error of estimation; RMSEcv, root mean square error of cross validation; SNV, standard normalised variate; MSC, multiplicative scatter correction.*

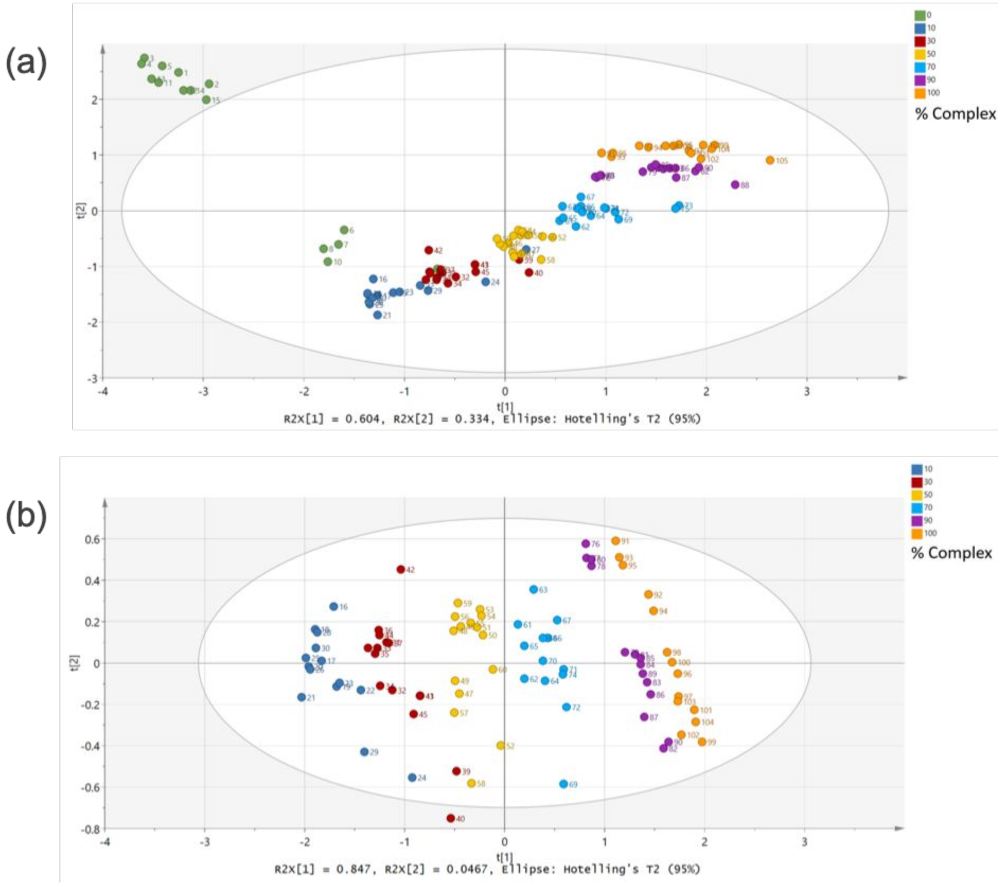

Supplementary Figure 4 Score plot of data after application of the first (a) and second (b) derivative filters, respectively, (t_1 versus t_2), containing two principal components. The plot was coloured according to percentage of the ABZ-MA 1:2 eutectic.

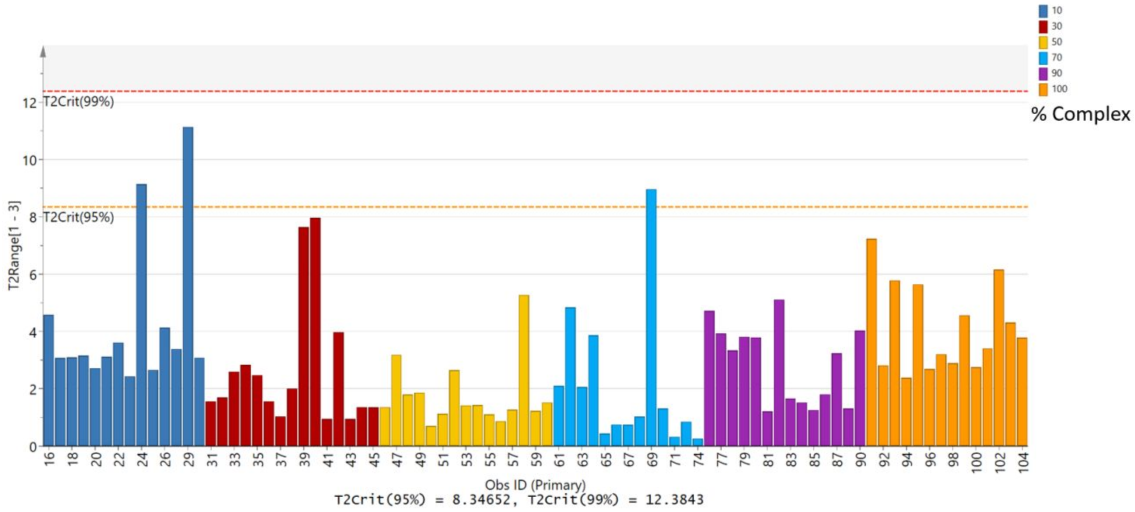

Supplementary Figure 5 Hotelling's T2 plot, showing three suspect outliers and no major outliers in the data after application of the second derivative filter. The 95 % and 99 % confidence limits are shown by the yellow and red dotted lines, respectively. The column plot was coloured according to percentage of the ABZ-MA 1:2 eutectic.

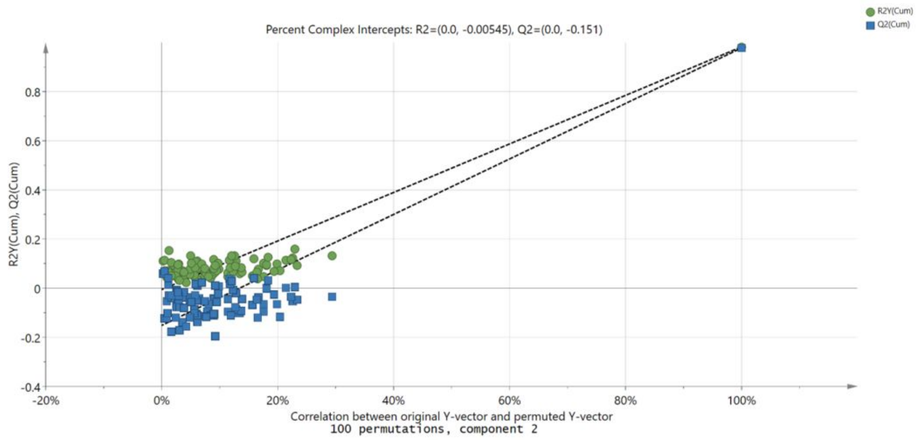

Supplementary Figure 6 Response permutation plot for validation of the model, where the green circles represent R^2Y and the blue squares Q^2. Q^2 intercepted at -0.151 and R^2 intercepted at -0.005.

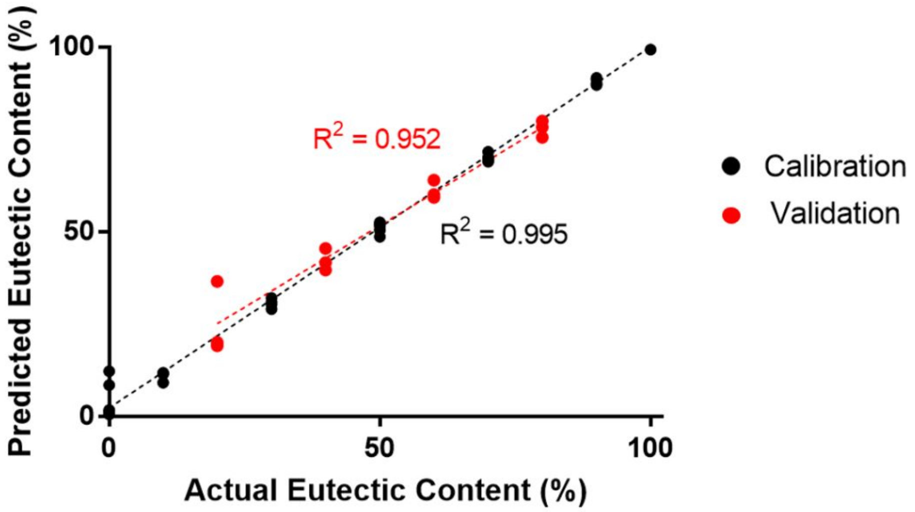

Supplementary Figure 7 Graph of observed versus predicted eutectic content (%).

Supplementary Table 2 Drug percentage (DP %) and dissolution rate (DR %/min) at particular timepoints for the EPO extrudates of ABZ and ABZ-MA 1:2.

	DP (%)			DR (%/min)		
	**5 min**	**30 min**	**120 min**	**5 min**	**30 min**	**120 min**
**ABZ-EPO**	7.1±1.2	17.7±0.5	38.8±0.9	1.4±0.2	0.6±0.1	$0.3±7.3x10^{-3}$
**ABZ-MA-EPO MiniLab**	8.7±0.6	22.3±0.7	68.9±1.3	1.7±0.1	0.7±0.02	$0.6±4.4x10^{-3}$
**ABZ-MA-EPO Rondol**	7.4±1.6	24.8±4.2	71.5±1.0	1.5±0.3	0.8±0.1	$0.6±8.3x10^{-3}$
